# A systematic review and narrative synthesis of physical activity referral schemes’ components

**DOI:** 10.1186/s12966-023-01518-x

**Published:** 2023-11-27

**Authors:** Eriselda Mino, Coral L. Hanson, Inga Naber, Anja Weissenfels, Sheona McHale, Jane Saftig, Sarah Klamroth, Peter Gelius, Karim Abu-Omar, Stephen Whiting, Kremlin Wickramasinghe, Gauden Galea, Klaus Pfeifer, Wolfgang Geidl

**Affiliations:** 1 Department of Sport Science and Sport, Friedrich-Alexander-Universität Erlangen-Nürnberg (FAU), Gebbertstraße 123B, 91058 Erlangen, Germany; 2 School of Health and Social Care, Edinburgh Napier University, Sighthill Campus, Edinburgh, EH11 4DN UK; 3 WHO European Office for Prevention and Control of Noncommunicable Diseases (NCD Office), Copenhagen, Denmark

**Keywords:** Physical activity, Physical activity referral scheme, Healthcare, Physical activity prescription, Exercise prescription

## Abstract

**Background:**

Physical activity referral schemes (PARS) are complex multicomponent interventions that represent a promising healthcare-based concept for physical activity (PA) promotion. This systematic review and narrative synthesis aimed to identify the constitutive components of PARS and provide an overview of their effectiveness.

**Methods:**

Following a published protocol, we conducted a systematic search of PubMed, Scopus, Web of Science, CINAHL, ScienceDirect, SpringerLink, HTA, Wiley Online Library, SAGE Journals, Taylor & Francis, Google Scholar, OpenGrey, and CORE from 1990 to January 2023. We included experimental, quasi-experimental, and observational studies that targeted adults participating in PARS and reported PA outcomes, scheme uptake, or adherence rates. We performed an intervention components analysis using the PARS taxonomy to identify scheme components and extracted data related to uptake, adherence, and PA behavior change. We combined these to provide a narrative summary of PARS effectiveness.

**Results:**

We included 57 studies reporting on 36 PARS models from twelve countries. We identified 19 PARS components: a patient-centered approach, individualized content, behavior change theory and techniques, screening, brief advice, written materials, a written prescription, referral, baseline and exit consultation, counselling support session(s), PA sessions, education session(s), action for non-attendance, structured follow-up, a PA network, feedback for the referrer, and exit strategies/routes. The PARS models contained a mean of 7 ± 2.9 components (range = 2–13). Forty-five studies reported PA outcome data, 28 reported uptake, and 34 reported adherence rates. Of these, approximately two-thirds of studies reported a positive effect on participant PA levels, with a wide range of uptake (5.7–100.0%) and adherence rates (8.5–95.0%).

**Conclusions:**

Physical activity referral scheme components are an important source of complexity. Despite the heterogeneous nature of scheme designs, our synthesis was able to identify 19 components. Further research is required to determine the influence of these components on PARS uptake, adherence, and PA behavior change. To facilitate this, researchers and scheme providers must report PARS designs in more detail. Process evaluations are also needed to examine implementation and increase our understanding of what components lead to which outcomes. This will facilitate future comparisons between PARS and enable the development of models to maximize impact.

**Supplementary Information:**

The online version contains supplementary material available at 10.1186/s12966-023-01518-x.

## Background

Chronic non-communicable diseases (NCDs) present a challenge to public health and modern healthcare systems [1]. Physical activity (PA) interventions offer a window of opportunity for NCD prevention and management, [2] particularly in primary care [3, 4]. This is because healthcare professionals are considered to be a credible source of information about the well-established health-enhancing benefits of PA [5]. In 2016, 39 billion outpatient healthcare visits were made globally [6], which, if utilized concurrently for PA promotion, might have reached an estimated 1.4 billion insufficiently inactive adults [7]. Physical activity healthcare interventions, such as brief advice and physical activity referral schemes (PARS), are considered viable approaches that enable healthcare professionals to encourage patients to be more active [4, 8, 9]. At the system level, PARS offer a practical way for healthcare professionals to harness the role of PA in reducing the burden of NCDs and help overcome fragmented efforts in PA promotion. At the individual level, referral schemes are suggested to improve not only PA of participants, but also their depression levels [10, 11], insulin sensitivity [12], body composition, and cardiometabolic risk factors [13]. Additionally, participants have reported a sense of belonging and social inclusion [14].

Physical activity referral schemes are widespread, complex interventions that involve the coordinated efforts of healthcare and exercise professionals in an individual’s journey to achieve PA behavior change. They are comparable to other healthcare referrals, which are defined as “the direction of an individual to the appropriate facility or specialist in a health system or network of service providers to address the relevant *health needs*” [4]. In PARS, individuals who have or are at risk of NCDs and have a *health need* in terms of insufficient PA are directed to appropriate PA specialists, facilities, or activities. These types of interventions offer an opportunity to break the ice between PA offers and inactive patients. As such, the World Health Organization advocates offering brief PA interventions, including referral pathways, in primary care to support PA behavior change [7]. Despite this endorsement, PARS have only demonstrated a modest impact on PA levels [15]. Current understanding of effectiveness is limited by the dominance of UK-based studies, which are characterized by high heterogeneity [16]. This has resulted in a lack of understanding about what works [17]. There is a need to better define different PARS models, so that reviews of evidence can distinguish between distinct designs (e.g., UK versus Swedish models). However, even with small individual-level effects, great benefits can be seen at the population level when interventions are disseminated at scale [18]. Thus, attention has been directed to embedding PARS into healthcare systems; for example, the European Physical Activity on Prescription model (EUPAP) project aims to establish the Swedish model in Belgium, Denmark, Germany, Italy, Lithuania, Malta, Portugal, Romania, and Spain [19].

Physical activity referral schemes incorporate various components to elicit behavior change [8, 20]. The Swedish model includes five components: a patient-centered approach, evidence-based PA recommendations, a written prescription, follow-up, and a community-based network [20, 21]. Schemes that incorporate these components are known to be effective, but it is unclear whether some components produce more favorable results than others [11]. Previous systematic reviews have called attention to PARS components [15], especially the component-effectiveness relationship [11] that is recognized as a researchable link in the complex intervention field [22, 23]. Complex intervention understanding and research can be approached by treating an intervention as a uniform package, “downplaying complexity,” or as an intervention composed of components, “recognizing complexity” [22]. At the systematic review level, PARS effectiveness has been examined as a complete package [10, 11, 15, 24], pooling only effect sizes and discounting intervention components. Other systematic reviews have explored PARS effectiveness in terms of scheme characteristics (referral reason and follow-up) [25, 26], but this is different from examining components. Components are single, active parts that comprise the entire PARS [22, 27] or guiding operational principles at scheme level [28], such as counseling using a patient-centered approach [20]. In contrast, PARS characteristics include setting, scheme length, and provider profession. While we acknowledge that complexity is multifaceted [29] and PARS characteristics may impact effectiveness [25], in this review, we have focused only on components as a source of complexity. The identification of components can enable the future investigation of their relative impact on effectiveness, creating useful knowledge for program developers and decision-makers [22, 29].

### Review question

As per our previously published protocol [28], we planned to examine PARS by reviewing the design of interventions to identify their constitutive components (Review Question 1) and further analyze their impact on effectiveness in terms of PA, uptake, and adherence (Review Question 2). In this paper, we focus on the first question by providing an overview of components that make up PARS models and information on their characteristics. Additionally, we present a narrative summary of the evidence of effectiveness.

## Methods

This systematic review was conducted by following the Cochrane Handbook for Systematic Reviews of Interventions [30] and reported by adhering to the Preferred Reporting Items for Systematic Reviews and Meta-Analyses (PRISMA) [31] and Synthesis without meta-analysis (SwiM) [32] guidelines. The methods were pre-registered in the protocol [28] and are briefly described here.

### Eligibility criteria

Eligible studies were those that investigated PARS initiated in a primary or secondary healthcare setting; targeted a population aged ≥ 16 years; and reported PA, uptake, or adherence outcomes. We considered all interventions labeled as PARS, exercise referral schemes, or exercise on prescription or any similar intervention, such as PA counselling that included at least some form of documentation, such as a prescription or referral form. Advice only, exercise/PA only, or combined lifestyle intervention studies that included other health behaviors in addition to PA were excluded. We included experimental, quasi-experimental, and observational studies that were published in English or German and reported the outcomes of interest, irrespective of the type of outcome measurement, methodological quality, comparison group, and follow-up duration.

### Search and study selection

We conducted systematic searches in Scopus, PubMed, Web of Science, CINAHL, ScienceDirect, SpringerLink, HTA, Wiley Online Library, SAGE Journals, Taylor & Francis, Google Scholar, OpenGrey, and CORE for articles published since 1990 (Additional file 1), combined with search methods such as citation and hand searching. The initial search was conducted by one author (EM) in June 2020 and updated on January 31, 2023 (Additional file 1). Duplicates were removed, and the remaining articles were downloaded into Citavi V.6 (Swiss Academic Software). Titles and abstracts were screened independently by one reviewer (EM) and a pair of reviewers (IN, AW). One reviewer (EM) screened all full texts. An independent second full-text screening was distributed among the team (AW, IN, JS). The extent of agreement was measured using Cohen’s kappa, and divergences were resolved via discussion.

### Data extraction and items

Reports on the same study were grouped together, and data on study characteristics, PARS content (characteristics and components), and effectiveness outcomes (PA, uptake, and adherence) were extracted. A single reviewer (EM) extracted the data into a customized Microsoft Excel spreadsheet (Microsoft Corporation, Washington, USA), with a second reviewer (JS) extracting 15% of included studies to check for accuracy.

#### Scheme content

Data were extracted at the scheme level using the PARS taxonomy, a classification system to document, audit, monitor, and report such programs [16]. We contacted twelve primary investigators to clarify questions or ask for support in the form of additional information, and half of them replied.

#### Effectiveness outcomes

We extracted total PA and also moderate to vigorous PA, leisure time PA, and walking when available. Additionally, we extracted scheme uptake and adherence rates. When the primary investigators did not explicitly define uptake or adherence, we extracted data that fit our predefined uptake definition, that is, attendance at the first PARS activity after receiving a referral or prescription or the extent to which the prescribed activities or enrolled programs were completed [28].

### Risk of bias in individual studies

This systematic review was solely focused on content analysis to identify PARS components (first review question [28]) and did not include a meta-analysis of the effects of components. A risk-of-bias assessment is not included in this review but is being prepared for a subsequent analysis related to the second review question, that is, which of the identified components has the potential to maximize scheme effectiveness in terms of PA level, uptake, and adherence rates [28].

### Synthesis methods

Data were synthesized following the principles of the first stage of intervention component analysis (ICA), which is intended to compare interventions in terms of their similarities and differences [33]. The first stage of ICA involves two parallel processes: (a) content analysis and (b) narrative effectiveness synthesis.

- We combined the inductive ICA approach to content analysis with a deductive approach using levels one and two of the PARS taxonomy, scheme classification, and characteristics [16]. The use of this taxonomy reduced the chances of the arbitrary identification of the components given that at least 43 experts from research, PARS provision, healthcare, and policy-making backgrounds were involved in its creation.
  Two authors (CLH and SM) conducted the content analysis, using NVIVO20 (QSR International, Melbourne, Australia) to organize the data. The analysis was checked by a third reviewer (EM). Given that PARS do not follow a standard design, we mapped the referral routes using cross-functional flowcharts in Lucidchart software [30] to aid in the comparison and identify patterns and structural components as per our protocol [23].

(b) Along with the identified components, effectiveness data were synthesized and presented in a tabular format. Physical activity outcomes were displayed by employing vote counting; that is, for each included study, we indicated the direction of the effect regardless of statistical significance [34]. Scheme uptake and adherence are given as percentages, as reported in the individual studies.

## Results

### Studies included

**Fig. 1:**
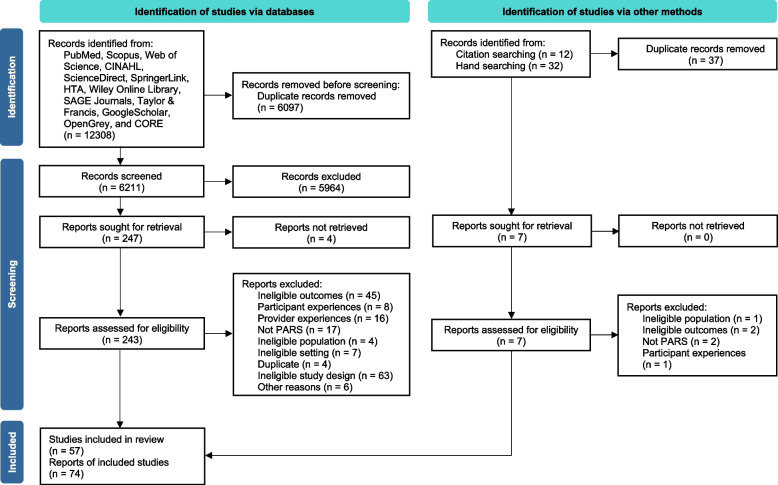
Study selection process

The systematic search of the databases yielded 6,211 unique records, and an additional seven were found through snowball searching (Fig. 1). We examined 243 full texts, and 74 met with this study’s eligibility criteria. Using the study as the unit of analysis [30], we conflated multiple reports of a single study, leading to 57 unique studies as the sample size for this systematic review. Reports of the same study presenting different outcomes (e.g., one reporting PA data and another reported adherence data) were included as separate study units ([35–38]). The extent of the agreement between reviewers for the inclusion of studies was strong (Cohen’s kappa = 0.804, 95% CI = 0.797–0.809).

### Study characteristics

The majority of studies (*n* = 28, 49.0%) used an experimental design (randomized controlled trial [RCT], pragmatic or cluster RCT) [39–67]. Sample sizes ranged from 14 [68] to 6,610 [69]. Studies were spread across four continents, and the most common location was Europe (*n* = 42, 73.7%) [35–46, 50, 51, 57, 58, 60, 61, 63, 64, 67–88]. Table 1 summarizes the study characteristics, and Additional file 2 describes them in more detail.

**Table 1:** Summary of characteristics of included studies

Characteristic	N (%)
Location	
UK	20 (35.0)
Sweden	12 (21.0)
New Zealand	6 (11.0)
Spain	3 (5.0)
Denmark	3 (5.0)
Canada	3 (5.0)
Australia	3 (5.0)
USA	2 (4.0)
Norway	1 (2.0)
The Netherlands	2 (4.0)
Mexico	1 (2.0)
Finland	1 (2.0)
Year of publication	
1990 to 2000	4 (7.0)
2001 to 2010	20 (35.0)
2011 to 2022	33 (58.0)
Study design	
Experimental design	28 (49.0)
Quasi-experimental design	12 (21.0)
Observational	17 (30.0)
Comparison group^1^	
Usual care	14 (25.0)
Advice only	5 (9.0)
Standard/low-intensive PARS	11 (19.0)
Prescription/referral only	3 (5.0)
Other comparison	4 (7.0)
No comparison	21 (37.0)
Sample size	
Total	44,690
Median, Range	316 (14 to 6610)
*14 to 250*	*27 (47.0)*
*251 to 1000*	*21 (37.0)*
> *1000*	*9 (16.0)*
Outcomes^1^	
Physical activity	45 (79.0)
*Subjective measure (e.g. questionnaires)*	*34 (75.6)*
*Objective measure (e.g. accelerometers)*	*4 (8.9)*
*Both*	*7 (15.6)*
Uptake	28 (49.0)
Adherence	34 (60.0)
Follow-up	
No follow-up (mid-, post-scheme)	23 (40.0)
1 to 6 months	20 (35.0)
7 to 12 months	13 (23.0)
24 months	1 (2.0)

^1^may be multiple per study, *PARS* physical activity referral scheme(s)

### Scheme characteristics

Table 2 summarizes the characteristics of the PARS models investigated in the included studies. More detailed information about each scheme (e.g., the content of the PA sessions) can be found in Additional file 4. The studies collectively investigated 36 PARS models, and seven schemes were researched by multiple studies. The Swedish Physical Activity on Prescription (PAP) model was investigated the most [35–38, 40–44, 58, 70–72, 89–91], with some studies examining schemes with the standard core components of this model [35–38, 41, 58, 72] and others focusing on enhanced variations [40, 42, 43, 70, 71]. The second most investigated model was the Green Prescription (GRx), originating from New Zealand, including the standard scheme [48, 62, 92] and variations [47, 53, 54]. This scheme was also replicated in the US [59]. Eighteen different schemes were included from the UK [45, 46, 50, 51, 63, 64, 68, 69, 73–83, 88]. These ranged from a simple referral to a PA program [81] to more complex referral systems [46].

**Table 2:** PARS characteristics

Characteristic	N
Length of scheme	
Open ended	12
8 weeks	1
10 weeks	4
12 weeks (3 months)	15
16 weeks (4 months)	6
18 weeks (5 months)	1
24 weeks (6 months)	7
36 weeks (9 months)	1
40 weeks (10 months)	2
12 months	6
15 months	1
24 months	1
Length of sessions	
30–45 min	1
45–60 min	11
Based on 150 min message	3
Not reported	40
No. of sessions per week	
1 × per week	4
2 × per week	10
3 × per week	6
PA Setting^1^	
Independent/Home-based	25
Local leisure facility, gym, sport clubs	34
Outdoor activity (walking, jogging, cycling)	2
Primary care	7
Unclear	1
Inclusion criteria	
Ability to pay	2
Motivation	2
Eligible conditions^1^	
*Cardiovascular disease*	*10*
*Cardiovascular risk factors*	*14*
*Coronary heart disease risk factors*	*6*
*Obesity, overweight*	*8*
*Metabolic syndrome*	*3*
*Diabetes mellitus*	*8*
*Falls prevention*	*2*
*Mental health conditions*	*10*
*Multiple sclerosis*	*2*
*Musculoskeletal*	*10*
*Neurological conditions*	*3*
*Respiratory*	*9*
*Cancer survivor*	*2*
*Unclear*	*1*
Cost to participants	21
Subsidized access to leisure centers	9
Referral to PARS	
Referral form emailed to PARS coordinator	1
Referral form faxed to PARS coordinator	2
Referral form posted to PARS coordinator	1
Referral form or prescription given to patient	7
Referral form or prescription unspecified process for transfer	11
Referral triage system	1
Unclear	34
Participant-provider initial contact	
Email contact	1
Mail contact	1
Telephone contact	5
Unspecified contact method	50
Referrer or prescriber`s profession^1^	
Exercise physiologist	1
Physician or general practitioner	45
Physiotherapist	12
Practice nurse	25
Other referrer	7
Primary healthcare professional, not specified	15
Secondary healthcare professional, not specified	7
Licensed healthcare professional, not specified	8
Self-referral	3
PA prescriber/provider qualifications	
Degree in sport science	1
National body accredited	4
UK level 3	6
UK level 4	1
PA qualification unspecified	45
Training for counseling and scheme processes^1^	14
Training about scheme process	11
Training for motivational interviewing	11
Training in behavior change techniques	1
Training in self-determination theory, social cognitive theory	5
Funding: External or internal funding	16

^1^may be multiple per study, *PARS* physical activity referral scheme(s), *PA* physical activity

### PARS components

The component analysis revealed 19 components that make up PARS (Table 3). While there was some inconsistency in the use of terms to designate intervention components, the definitions that were established during the analysis can be found in Additional file 3.

**Table 3:** Components identified in PARS

Component	N (%)
Person-centered approach	29 (50.9)
Individualized content	45 (78.9)
Behavior change theory^1^	26 (45.6)
Social cognitive theory	9 (34.6)
Self-determination theory	5 (19.2)
Transtheoretical model	16 (61.5)
Behavior change techniques^1^	37 (64.9)
Goal setting, action planning	28 (77.8)
Review of behavior/outcome goal(s)	9 (25.0)
Information about health/emotional consequences	12 (33.3)
Self-monitoring of behavior	11 (30.5)
Problem solving, coping planning, relapse prevention	18 (48.6)
Reduce negative emotions, focus on past success	2 (5.5)
Feedback on behavior, positive reinforcement	4 (11.1)
Prompts/cues	2 (5.5)
Graded tasks	1 (2.8)
Restructuring the physical environment	1 (2.8)
Social support	10 (27.8)
Screening	26 (45.6)
Identified during routine visits	21 (80.8)
Participant information in waiting room	1 (3.8)
Patient prompted the healthcare professional	1 (3.8)
Physical activity screening tool	1 (3.8)
Brief advice	17 (29.8)
Written materials	11 (19.3)
Written prescription	37 (64.9)
Referral to PARS program/professional	31 (54.4)
Baseline consultation	34 (59.6)
Exercise or community professional led consultation	22 (64.7)
Healthcare professional led consultation	12 (35.3)
Includes fitness assessment and counseling	6 (17.6)
Only behavioral counseling	23 (67.6)
Only fitness assessment	3 (8.8)
Unclear	2 (5.9)
Exit consultation	23 (40.4)
Includes fitness assessment and counseling	5 (21.7)
Only behavioral counseling	15 (65.2)
Only fitness assessment	2 (8.7)
Unclear	1 (4.3)
Counseling support session(s)	24 (42.1)
In person support	11 (45.8)
Telephone support	1 (4.2)
Web-based support	1 (4.2)
Varying levels of support	7 (29.2)
Not specified	4 (16.6)
PA sessions	26 (45.6)
Activities included^1^	
*Aerobic activity*	11 (42.3)
*Anaerobic activity*	2 (7.7)
*Chair based*	2 (7.7)
*Dancing*	2 (7.7)
*Games*	3 (11.5)
*Group based classes*	19 (73.1)
*Mobility and flexibility*	5 (19.2)
*Racquet sports*	3 (11.5)
*Strength activity*	11 (42.3)
*Tai-chi and yoga*	2 (7.7)
*Type not specified or mixed*	8 (30.8)
*Walking*	5 (19.2)
*Water-based*	9 (34.6)
Education session(s)	3 (5.3)
Action for non-attendance	7 (12.3)
Structured follow-up	14 (24.6)
PA network	9 (15.8)
Feedback to referrer	4 (7.0)
Exit routes/strategies	19 (33.3)

^1^may be multiple per study, *PARS* physical activity referral scheme(s), *PA* physical activity

The identified components appertain to the following:

- the theoretical basis (person-centered approach, individualized content, and behavior change theory and techniques);
- scheme entry and transitioning and exit (screening, brief advice, written prescription, referral, exit routes/strategies, and feedback to the referrer);
- behavioral support (baseline consultation, final consultation, counseling support session(s), structured follow-up, action for non-attendance, education session(s), and written materials);
- and PA opportunities (PA sessions and a PA network).

For some of the components, we were able to identify specific elements that are listed in Table 3, together with frequencies.

There was substantial variation in the number of components included within the design of various PARS. The PARS models contained a mean of 7 ± 2.9 components (range = 2–13).

### Narrative effectiveness synthesis

Table 4 summarizes the distribution of the 19 components across the 57 studies. For each study, the components are indicated as present or not and mapped against the effect direction on PA level (regardless of significance level), uptake rate, and adherence rate. These data are solely descriptive and are not intended to indicate the effectiveness of specific components.

**Table 4:** PARS components and PA effectiveness data

PARS model	Person-centered approach	Individualized content	Behavior change theory	Behavior change techniques	Screening	Brief advice	Written materials	Written prescription	Referral	Baseline consultation	Exit consultation
**PARS vs usual care**											
Prex, Finland [39]	I	I	I	I	I	I		I			
PAP, Sweden [41]	I	I	I	I	I			I		I	
PAP, Sweden [58]	I	I		I				I		I	
Enhanced GRx, NZ [47] ([93]‡)	I	I		I	I	I		I			
GRx, NZ [48] ([94, 95]‡)	I	I		I	I	I	I	I			
HLC model, Norway [61]	I	I	I	I					I	I	I
NERS, UK [46] ([96, 97]‡)	I	I		I	I				I	I	I
Exercise referral programme, UK [63]									I	I	I
EoP, Netherlands [67]				I				I	I	I	I
Referral to AEP, Australia [52] ([98]‡)	I	I	I	I	I			I	I	I	
ENGAGE, Australia [56]		I	I	I					I	I	
Active Practice, Australia [99]			I				I	I			
ERS, Spain [57]				I					I		
PAP+referral, Canada [100]			I			I		I	I		
**PARS vs advice**											
Enhanced PAP, Sweden [40]	IC	IC		IC	IC		IC	I		IC	
ERS, Mexico [49] ([101, 102]‡)		I	I	I	IC	IC		I	I	I	
Fitness for Life, UK [51]			I			C	C		I	I	I
‘Walking Partners’ scheme, UK [51]						C	C		IC		
GRx, USA [59]				IC		IC		I			
GRx, New Zealand [62]				IC		IC		I			
**PARS vs. prescription**											
PAP+referral, Canada [100]						IC		IC	I		
Majorca model, Spain [60]	I	I	I	I			I	IC		I	I
ERS, UK [50]		I					IC		IC	I	I
**Enhanced vs. standard / High-dose vs low-dose PARS**											
Co-PARS, UK [73]	I	I	I	I					IC	IC	IC
Enhanced PAP, Sweden [42] ([90, 103]‡)	IC	IC	I	I	IC	IC		IC			
IPAC, Canada [55]	IC	I	IC	IC	IC	IC		IC		I	I
ERS + e-coachER¹, UK [64] ([104, 105]‡)			I	I					IC		
STEPS, Canada [65]		IC	I					IC		I	
PAP enhanced, Sweden [43] ([91]‡)	IC	IC	I	I	IC		C	IC	I	I	I
EoP, Denmark [44]	IC	IC	IC	IC	IC			IC	IC	IC	IC
EoP, Denmark [85] ([106]‡)	IC	IC	IC	IC	IC	IC		IC	I	IC	IC
Birmingham EoP scheme, UK [45]	IC	IC	I	IC			I	IC		IC	IC
PAP with CS, Sweden [70]	IC	IC				IC		IC			
Pedometer step-based GRx, New Zealand [53] ([107]‡)	IC	IC	IC	IC	IC	IC		IC			
**Other comparison**											
GRx, New Zealand [92]		IC		IC				IC			
GRx, New Zealand [54]	IC	IC		IC	IC		IC	IC			
EoP, USA [66]		IC		IC	IC			IC	IC	IC	
Active Lifestyle ERS [88]		IC		IC				IC	IC	IC	IC
**No comparison group**											
PAP, Sweden [35, 36]	I	I	I	I	I	I		I			
Östergötland PARS, Sweden [37, 38] ([89])	I	I			I	I		I			
PAP, Sweden [71]	I	I		I	I		I	I		I	I
EoP, Denmark [84]	I	I	I	I	I			I		I	I
Northumberland ERS, UK [75]		I	I	I					I	I	I
Northumberland ERS, UK [76]		I	I	I					I	I	I
Scottish PARS, UK [77]									I		
PAFES PARS, Spain [86]					I				I		
ERS Tameside, UK [78]		I							I	I	I
PAP, Sweden [72]	I	I		I	I			I		I	
Active Living for Life, UK [79]		I							I	I	I
Heartlinks, UK [80]	I	I	I	I			I		I	I	I
NERS – exercise referral only, UK [68]		I							I		
Proactive scheme, UK [81] ([108]‡)									I		
Birmingham EoP scheme, UK [74]	I	I	I	I			I	I		I	I
ERS, UK [69]		I					I		I	I	I
Stockport EoP scheme, UK [82]		I			I			I		I	
ERS, UK [83]		I			I				I		
Referral to peer coach PA, The Netherlands [87]									I		

PARS model	Counseling support session(s)	PA sessions	Education session(s)	Action for non-attendance	Structured follow-up	PA network	Feedback to referrer	Exit strategies/routes	PA effect direction	PARS uptake	Adherence
**PARS vs usual care**											
Prex, Finland [39]					I				+	n/a	n/a
PAP, Sweden [41]	I		I		I	I			+	n/a	n/a
PAP, Sweden [58]					I				ND	n/a	n/a
Enhanced GRx, NZ [47] ([93]‡)	I				I		I		+	98.0%†	95.0% received full intervention†
GRx, NZ [48] ([94, 95]‡)					I				+	65.5 %	85.0%
HLC model, Norway [61]		I							ND	n/a	73.7 %
NERS, UK [46] ([96, 97]‡)	I	I		I	I			I	+	85.1 %	85% (43.8 % fully, 41.33 % partly)
Exercise referral programme, UK [63]		I							ND/+	87.6%	28.0% (at least 15/20 sessions), 41.0% (at least 10/20), 68.0% (at least 5/20)
EoP, Netherlands [67]	I	I		I				I	+	99.0%†	86.0%
Referral to AEP, Australia [52] ([98]‡)	I								+	91.7%†	76.0% fully adhered 85.0% attended at least 4/5 sessions
ENGAGE, Australia [56]		I						I	ND/+	n/a	85.0%
Active Practice, Australia [99]									+	n/a	n/a
ERS, Spain [57]		I						I	+	n/a	71%
PAP+referral, Canada [100]	I								+	n/a	n/a
**PARS vs advice**											
Enhanced PAP, Sweden [40]	I		I		I				ND	n/a	n/a
ERS, Mexico [49] ([101, 102]‡)		I						I	ND	78.6%	78.0% attended ≥ 50.0% of the sessions
Fitness for Life, UK [51]		I						I	ND/+	92.0%	82.0% attended at least 25.0% of sessions, 42.0% high-adherence
‘Walking Partners’ scheme, UK [51]		I						I	+	76.5%	56.0% attended at least 25.0% of sessions, 21.5% high-adherence
GRx, USA [59]									ND	n/a	n/a
GRx, New Zealand [62]									+	n/a	n/a
**PARS vs. prescription**											
PAP+referral, Canada [100]									ND	n/a	n/a
Majorca model, Spain [60]	I					I			ND/+	n/a	n/a
ERS, UK [50]								I	+	n/a	n/a
**Enhanced vs. standard / High-dose vs low-dose PARS**											
Co-PARS, UK [73]	I							I	ND	85.0%†	71.4%, 60.7% and 32.1% attended 3rd, 4th and 5th consultation session out of 5 respectively
Enhanced PAP, Sweden [42] ([90, 103]‡)	I				IC	IC			ND	n/a	57.0%
IPAC, Canada [55]	I							I	+/ND	88.0% for I&C	87.0%
ERS + e-coachER¹, UK [64] ([104, 105]‡)	I								+	75.0% I, 78.0% C	n/a
STEPS, Canada [65]									+	n/a	n/a
PAP enhanced, Sweden [43] ([91]‡)		I						I	ND	48.0%†	46.6%
EoP, Denmark [44]	IC	I	I					I = 1 C = 0	ND	100%†	Exercise sessions attendance (I): 18/24 (q1, 14.8; q2, 21.3) Counseling attendance: I=76.0%, C=91.0%
EoP, Denmark [85] ([106]‡)	IC	I						I	ND	n/a	n/a
Birmingham EoP scheme, UK [45]				IC	I			IC	ND	n/a	n/a
PAP with CS, Sweden [70]	I								+	37.0%	n/a
Pedometer step-based GRx, New Zealand [53] ([107]‡)	IC				IC				ND/+	n/a	84.0%
**Other comparison**											
GRx, New Zealand [92]	I	I			C				+	31.9%	I=8.5%, C=24.4%
GRx, New Zealand [54]	IC								n/a	100%	n/a
EoP, USA [66]		IC							n/a	n/a	26.35 (SD = 10.85) sessions
Active Lifestyle ERS [88]	IC	IC						IC	n/a	88.0% I, 68.9% C	Weekly adherence: I = 48% ± 35, C = 39% ± 36
**No comparison group**											
PAP, Sweden [35, 36]						I			+ [32]	n/a	65.0% adhered to PAP, 19.0% partly adhered, 16.0% non-adherence [33]
Östergötland PARS, Sweden [37, 38] ([89])				I	I	I			+[34]	n/a	56.0% at 3 months, 50.0% at 12 months [35]
PAP, Sweden [71]	I								+	n/a	n/a
EoP, Denmark [84]	I	I						I	+	n/a	n/a
Northumberland ERS, UK [75]	I	I					I	I	+	75.7%	40.0%†
Northumberland ERS, UK [76]	I	I		I					+	81.0%	53.5% attended mid-scheme, 42.9% completed the scheme
Scottish PARS, UK [77]		I							+/ND	83.8%	43.0%
PAFES PARS, Spain [86]		I							+	n/a	75.0% completed, 84.1% average attendance
ERS Tameside, UK [78]	I	I							+	76.5%	38.7% completed
PAP, Sweden [72]					I	I			+	n/a	n/a
Active Living for Life, UK [79]									+	64.5%	47.4% completed
Heartlinks, UK [80]	I	I					I		+	n/a	45.5% completed
NERS – exercise referral only, UK [68]		I							+	n/a	78.6%
Proactive scheme, UK [81] ([108]‡)		I				I			n/a	68.8%	48.3%
Birmingham EoP scheme, UK [74]				I	I		I	I	n/a	n/a	51.0%
ERS, UK [69]								I	n/a	79.0%	n/a
Stockport EoP scheme, UK [82]		I							n/a	60.0%	30.6%
ERS, UK [83]		I			I			I	n/a	89.3%	82.4% completed stage one 51.8% continued stage two
Referral to peer coach PA, The Netherlands [87]		I							n/a	5.7%	66.7%

Abbreviations: *n/a* Not available, *SC* Counseling support, *Prex* Prescription-based PA counseling, *PAP* Physical Activity on Prescription, *GRx* Green Prescription, *HLC* Healthy Lifestyle Centers, *NERS* The Welsh National Exercise Referral, *Co-PARS* Coproduced PA Referral Scheme, *EoP* Exercise on Prescription, *AEP* Accredited Exercise Physiologists, *ERS* Exercise Referral Scheme, *IPAC* Intensive Physical Activity Counseling, *STEPS* Step Test Exercise Prescription Stage of Change Counseling, *PARS* Physical Activity Referral Scheme

I, The component is present only in the intervention group

C, The component is present only in the comparison group

IC, The component is present in both the intervention and comparison group

+ , PA level increased regardless of statistical significance

*ND* No difference between groups or pre-post for studies without a comparison group

^†^, Our calculation/interpretation of data

^‡^, Report of the same study, but no data extracted. Reports from the same study, merged with the included study that represents the main data source

^1^, Web-based behavioral support is classified under the component “counseling support session(s).” The participants in both groups receive one of three typical ERS models that differ from one another. The only difference between groups is the web-based behavioral support intervention added to the intervention group. Thus, the components rating provided here are made based only on this added intervention

The majority of studies reported positive effects on the part of PARS on PA levels [39, 41, 46–48, 52, 57, 67, 99, 100] as compared with usual care, while four RCTs reported no group difference [58, 61] or mixed results [56, 63]. In contrast, only one randomized trial reported any additional benefit on the part of PARS on PA level [62] when compared with PA advice alone, while three trials did not detect any additional benefit [40, 49, 59], and one reported mixed results [51]. The offer of a PARS program was shown to be more beneficial in terms of increasing PA than prescription only [50, 100], with inconsistent results being found in one study [60]. Approximately one-fifth of the included studies compared different versions of PARS regarding intensity and the activities offered. Most studies did not report added benefits for an enhanced intervention over standard provisions [42–45, 73, 85]. However, two trials [64, 65] and one observational study [70] reported that more intensive PARS offer added benefits for participants, and one study reported inconsistent results [55]. Observational and pre-post studies consistently reported an increase in PA levels for PARS participants [35, 37, 68, 71, 72, 75, 76, 78–80, 84, 86], with the exception of one study [77].

Among the 28 studies that reported uptake, rates ranged from 5.7% [87] to 100.0% [44, 54]. Although not always explicitly stated, the uptake definition was consistent among studies, i.e., the number of participants who entered the scheme after being referred to. In other words, those who participated in at least one scheme activity after the referral. The adherence or attendance rate was reported in 34 studies, with variations in terms of definitions. For example, adherence was defined as adherence to the prescribed PA, adherence to the allocated PARS intervention, scheme completion, or the average attended PA sessions. Adherence rates varied from 8.5% [92] to 95.0% in terms of completing the entire PARS [47].

## Discussion

This is the first review to examine the components that are included in PARS. We identified 19 components: using a person-centered approach, individualized content, being based on behavior change theory, the use of BCTs, screening, brief advice, the provision of written materials, written prescriptions, referral to a PARS program/professional, a baseline consultation, an exit consultation, counseling support session(s), PA sessions, education session(s), action for non-attendance, structured follow-up, PA networks, feedback to the referrer, and having exit routes/strategies. The PARS models we examined contained a mean of 7 ± 2.9 components (range = 2–13). The level of detail provided in studies of PARS content varied, making it difficult to ensure that all components were identified. In our narrative effectiveness synthesis, approximately two-thirds of studies reported a positive effect on participant PA levels, with wide ranges of uptake (5.7–100.0%) and adherence rates (8.5–95.0%). The large cross-country and within-country (for example, UK) differences in the number and arrangement of components included in the PARS models in this review highlights the complexity of understanding which components affect which outcomes. This is not only because these differences might impact effect sizes (changes in PA) and participant engagement with the scheme (uptake and adherence). The inclusion of different components in a scheme creates differing implementation demands, which must be adequately resourced. Implementation fidelity will be reflected in scheme outcomes, adding another layer of complexity.

The complexity of the role of components within PARS has played a limited role in evidence synthesis to date. Existing PARS meta-analyses have synthesized the effects of PARS interventions as an uniform package [10, 15], without any consideration of differences in design and delivery. Thus, the true heterogeneity of PARS models, as a function of their components, has not been incorporated in the effectiveness equation. Previous reviews have considered the potential influence of demographics (e.g., age, sex, and socio-economic status) [109, 110], personal factors (e.g., referral reasons, medical conditions, and psychological factors) [14, 109, 110], healthcare system/team-related factors (e.g., adequacy of health services and participant-provider relationship) [110], and scheme characteristics (e.g., scheme length, number of exercise sessions, and scheme setting) [25, 109] on uptake and adherence rates, as well as PA behavior change. Our findings advance the prior understanding of PARS complexity by highlighting specific scheme components (e.g., brief advice and PA sessions), in addition to other relevant demographic or personal factors.

The reviewed evidence demonstrates that single PARS components are a subject of growing interest, but they have not been included in meta-analyses. Many of the included studies have the potential added effect of certain components, such as behavior change theory [45, 65], a written prescription [59, 62], written materials [99], counseling support [42, 55, 64, 70], and PA sessions [49], on PA and health outcomes. Additionally, components such as individualization [40, 42, 44], exit routes and strategies [74, 75], measures to keep scheme participation high [77], baseline consultation [77], and structured follow-up [51, 75, 99] have been suggested to be important to scheme success. This growing attention to the role of components in individual studies, in combination with heterogeneous scheme designs, risks producing research that is difficult to combine for synthesis. Our review highlights the fact that there is not yet a standard terminology that can be used to understand these differences between PARS designs. Our analysis adds value because it has distinguished between PARS components and provides a basis for a future standardized terminology. This will aid in scheme comparison and allow for evidence harmonization and synthesis. To enable better differentiation between PARS and an examination of which components add value, researchers and providers must improve the reporting of scheme content.

A lack of detailed information on intervention content and other study-relevant items is a known problem, despite the widespread recommendations of reporting guidelines [111], and this is reflected in the findings of this review. The incomplete reporting of behavioral interventions has a direct impact on identifying and understanding how intervention characteristics actually impact behavior [112]. Therefore, we suggest using the PARS checklist [16] to provide sufficient data quantity and clear information in a standardized way. Given the review findings, it may be beneficial to extend the checklist to include a section about counseling support session(s) and how these are offered. The PARS checklist [16] can be employed directly at the protocol stage, as utilized in one of our ﻿projects [113], or as a compass when designing interventions. Differentiating between scheme components strengthens comparability at the scheme level and can facilitate future research endeavors.

Studies show that individual components may have potential to maximize PARS effectiveness [62, 65, 70, 99]. This is important given the ambiguity in the existing evidence regarding the effect of PARS on PA and other health outcomes [8, 18]; thus, we strongly recommend the further investigation of the role of components in order to improve the case for investment in PARS. While we have identified potential components, their role in the effectiveness equation depends on their successful implementation. Only if the component under study is delivered as intended can its relevance to scheme success be determined. Thus, process evaluations of PARS [97, 104] are essential to understanding components.

### Strengths and weaknesses

The strength of this systematic review lies in the prior publication of the protocol [28], which reduced the chance of bias. We used a comprehensive search strategy, involving independent reviewers in the selection of studies for inclusion and using a standardized synthesis process in the identification of components. Additionally, the use of ICA [33] in combination with the PARS taxonomy [16] allowed for a systematic assessment of the intervention content of 36 models.

The results of the component analysis are, however, bound by two unique limitations. Both are closely related to the identification of the components and the rating of schemes as having or not having these components. Firstly, because the identification of PARS components was partially subjective, confirmation bias cannot be ruled out. Thus, the components list is by no means exhausting, and we may have overlooked other potentially relevant components. Secondly, poor reporting may have compromised our ability to detect certain components within a PARS when they were, in fact, present. The reporting level of the included studies varied substantially, from very detailed (e.g., [60, 73]) to a scant description of PARS content (e.g., [79]).

The terminology used to label components was inconsistent. Thus, during the ICA, the rating of a component as present or absent was based on its content, rather than the original label provided by the primary investigators. The identified components might also overlap with one another. For instance, individualization can be an inherent part of a person-centered approach, but one can individualize the content of PA sessions in an arbitrary way, without actively involving the participant in the process. Thus, we separated the concepts of person-centeredness and individualization, although they were often conflated in individual studies. One can also argue that a specific BCT, such as goal setting, could be a separate [114] of PA interventions. However, we focused on scheme-level components, that is, whether BCTs were incorporated. We applied the same reasoning for behavior change theory. While a particular type of theory can impact the intervention effects, the question of whether a PARS being theory-based impacts the PARS outcomes is more relevant to this review.

## Conclusions

Physical activity referral scheme components are an important source of complexity, and this review identified 19 components included in 36 PARS models that were delivered in twelve countries. Further research is required to determine the influence of these components on PARS uptake, adherence, and PA behavior change. To facilitate this, we recommend that researchers and scheme providers report PARS designs in more detail. We also suggest the need for process evaluations to examine the implementation of PARS designs and the role of components. This will increase our understanding of what works, leading to increased scheme optimization.

### Supplementary Information

**Additional file 1.** Search strategy results. This file contains the systematic search strategy and results for all the literature databases. **Additional file 2.** Overview of included studies sorted by comparison group. This file contains the characteristics of all the studies included in the systematic review, including the main results. **Additional file 3.** Description of PARS components. This file contains the description of the nineteen components identified through the content analysis. **Additional file 4.** Overview of PARS characteristics sorted by country. This file contains the characteristics of PARS models included in the systematic review. **Additional file 5.** PARS identified worldwide. This file provides an overview of all the PARS models identified during the screening for eligible articles, including those that we were not able to include in this review.

## Data Availability

All data relevant to the results of this systematic review are available in the main tables and additional files. The data collected, including data extraction forms, can be made available upon reasonable request.

## Abbreviations

BCTs: Behavior change techniques
ICA: Intervention component analysis
NCDs: Non-communicable diseases
PA: Physical activity
PAP: Physical activity on prescription
PARS: Physical activity referral scheme(s)
PRISMA: Preferred reporting items for systematic review and meta-analysis
RCT: Randomized controlled trial
SwiM: Synthesis without meta-analysis
