# Supplementary material for: A systematic review and narrative synthesis of physical activity referral schemes’ components
Source: Int J Behav Nutr Phys Act. 2023 Nov 27;20:140. doi: 10.1186/s12966-023-01518-x (PMC10683187; doi:10.1186/s12966-023-01518-x)
Supplement: Supplementary file 1 — Additional file 1. Search strategy results. This file contains the systematic search strategy and results for all the literature databases. Additional file 2. Overview of included studies sorted by comparison group. This file contains the characteristics of all the studies included in the systematic review, including the main results. Additional file 3. Description of PARS components. This file contains the description of the nineteen components identified through the content analysis. Additional file 4. Overview of PARS characteristics sorted by country. This file contains the characteristics of PARS models included in the systematic review. Additional file 5. PARS identified worldwide. This file provides an overview of all the PARS models identified during the screening for eligible articles, including those that we were not able to include in this review. [file 12966_2023_1518_MOESM1_ESM.docx]

Additional files

A systematic review of physical activity referral schemes’ components

| Contents |  | Pages |
| --- | --- | --- |
| Additional file 1: Search strategy results. This file contains the systematic search strategy and results for all the literature databases. |  | 2-8 |
| Additional file 2: Overview of included studies sorted by comparison group. This file contains the characteristics of all the studies included in the systematic review, including the main results. |  | 9-18 |
| Additional file 3: Description of PARS components. This file contains the description of the nineteen components identified through the content analysis. |  | 19 |
| Additional file 4: Overview of PARS characteristics sorted by country. This file contains the characteristics of PARS models included in the systematic review. |  | 20-28 |
| Additional file 5: PARS identified worldwide. This file provides an overview of all the PARS models identified during the screening for eligible articles, including those that we were not able to include in this review. |  | 29 |
| References |  | 30 |

Additional file 1. Search strategy results

| **PubMed** (19.06.2020; updated on 31.01.2023) | | | | | |  |
| --- | --- | --- | --- | --- | --- | --- |
| **Nr.** | | | **Query string** | **Hits** | |  |
|  | | | ***Stage one*** |  | |  |
| #1 | | | exercise referral scheme*[Title/Abstract] | 94 | |  |
| #2 | | | exercise referral [Title/Abstract] | 118 | |  |
| #3 | | | exercise prescription schemes [All Fields] | 55 | |  |
| #4 | | | exercise on prescription[Title/Abstract] | 32 | |  |
| #5 | | | physical activity prescription[Title/Abstract] | 100 | |  |
| #6 | | | physical activity referral scheme*[Title/Abstract] | 13 | |  |
| #7 | | | green prescription[Title/Abstract] | 41 | |  |
| #8 | | | physical activity prescription scheme[All Fields] | 58 | |  |
| #9 | | | physical activity referral[Title/Abstract] | 21 | |  |
|  | | | *all above 532 without duplicates makes 334* |  | |  |
| **#10** | | | #1 OR #2 OR #3 OR #4 OR #5 OR #6 OR #7 OR #8 OR #9 | 334 | |  |
|  |  |  | ((((((((exercise referral scheme*[Title/Abstract]) OR (exercise referral[Title/Abstract])) OR (exercise prescription schemes)) OR (exercise on prescription[Title/Abstract])) OR (physical activity prescription[Title/Abstract])) OR (physical activity referral scheme*[Title/Abstract])) OR (green prescription[Title/Abstract])) OR (physical activity prescription scheme)) OR (physical activity referral[Title/Abstract]) |  | |  |
|  | | | *after filters applied* | **333** | |  |
|  | | | ***Stage two*** |  | |  |
| #1 | | | motor activity[MeSH Terms] | 288.876 | |  |
| #2 | | | physical activit*[Title/Abstract] | 112.616 | |  |
| #3 | | | Exercise[MeSH Terms] | 193.955 | |  |
| #4 | | | exercise therapy[MeSH Terms] | 50.416 | |  |
| #5 | | | exercise[Title/Abstract] | 263.783 | |  |
| **#6** | | | #1 OR #2 OR #3 OR #4 OR #5 | 520.87 | |  |
| #7 | | | counseling[MeSH Terms] | 43.757 | |  |
| #8 | | | referral and consultation[MeSH Terms] | 74.52 | |  |
| #9 | | | directive counseling[MeSH Terms] | 4.135 | |  |
| #10 | | | Prescriptions[MeSH Terms] | 35.325 | |  |
| #11 | | | prescription[Title/Abstract] | 80.178 | |  |
| #12 | | | (referral[Title/Abstract]) OR (prescribed physical activity[Title/Abstract]) | 97.473 | |  |
| **#13** | | | #7 OR #8 OR #9 OR #10 OR #11 OR #12 |  | |  |
|  | | | (((((counseling[MeSH Terms]) OR (referral and consultation[MeSH Terms])) OR (directive counseling[MeSH Terms])) OR (Prescriptions[MeSH Terms])) OR (prescription[Title/Abstract])) OR ((referral[Title/Abstract]) OR (prescribed physical activity[Title/Abstract])) | 293.666 | |  |
| #14 | | | primary health care[MeSH Terms] | 158.363 | |  |
| #15 | | | health promotion[MeSH Terms] | 76.456 | |  |
| #16 | | | physicians, primary care[MeSH Terms] | 3.421 | |  |
| #17 | | | general practi*[Title/Abstract] | 82.089 | |  |
| #18 | | | primary care intervention*[Title/Abstract] | 455 | |  |
| **#19** | | | #14 OR #15 or #16 OR #17 OR #18 |  | |  |
|  | | | ((((primary health care[MeSH Terms]) OR (health promotion[MeSH Terms])) OR (physicians, primary care[MeSH Terms])) OR (general practi*[Title/Abstract])) OR (primary care intervention*[Title/Abstract]) | 305.387 | |  |
| **#20** | | | #6 AND #13 AND #19 | 1.872 | |  |
|  | | | ((((((motor activity[MeSH Terms]) OR (physical activit*[Title/Abstract])) OR (Exercise[MeSH Terms])) OR (exercise therapy[MeSH Terms])) OR (exercise[Title/Abstract])) AND ((((((counseling[MeSH Terms]) OR (referral and consultation[MeSH Terms])) OR (directive counseling[MeSH Terms])) OR (Prescriptions[MeSH Terms])) OR (prescription[Title/Abstract])) OR ((referral[Title/Abstract]) OR (prescribed physical activity[Title/Abstract])))) AND (((((primary health care[MeSH Terms]) OR (health promotion[MeSH Terms])) OR (physicians, primary care[MeSH Terms])) OR (general practi*[Title/Abstract])) OR (primary care intervention*[Title/Abstract])) |  | |  |
|  | | | *after filters applied* | 1815 | |  |
|  | | | **Remaining** | **2158** | |  |
|  | | | **Updated on 31.01.2023** | **748** | |  |
| **Scopus** (19.06.2020; updated on 31.01.2023) | | | | | | |
| **Nr.** | | **Query string** | | **Hits** | | |
|  | | ***Stage one*** | |  | | |
| #1 | | TITLE-ABS-KEY ( "exercise referral scheme*") | | 109 | | |
| #2 | | TITLE-ABS-KEY ( {exercise referral} ) | | 144 | | |
| #3 | | TITLE-ABS-KEY ( {exercise referral schemes} ) | | 73 | | |
| #4 | | TITLE-ABS-KEY ( {exercise on prescription} ) | | 52 | | |
| #5 | | TITLE-ABS-KEY ( {physical activity prescription} ) | | 116 | | |
| #6 | | TITLE-ABS-KEY ( "physical activity referral scheme*" ) | | 20 | | |
| #7 | | TITLE-ABS-KEY ( {green prescription} ) | | 53 | | |
| #8 | | ALL ( {physical activity prescription scheme} ) | | 0 | | |
| #9 | | TITLE-ABS-KEY ( {physical activity referral} ) | | 28 | | |
| **#10** | | #1 OR #2 OR #3 OR #4 OR #5 OR #6 OR #7 OR #8 OR #9 | | 367 | | |
|  | | TITLE-ABS-KEY ( "exercise referral scheme*" ) OR TITLE-ABS-KEY ( {exercise referral} ) OR TITLE-ABS-KEY ( {exercise referral schemes} ) OR TITLE-ABS-KEY ( {exercise on prescription} ) OR TITLE-ABS-KEY ( {physical activity prescription} ) OR TITLE-ABS-KEY ( "physical activity referral scheme*" ) OR TITLE-ABS-KEY ( {green prescription} ) OR ALL ( {physical activity prescription scheme} ) OR TITLE-ABS-KEY ( {physical activity referral} ) | |  | | |
|  | | *after filters applied (didn't import duplicates: only 97 new) (Status citavi: 2103)* | | **364** | | |
|  | | ***Stage two*** | |  | | |
| #1 | | INDEXTERMS ( {Motor Activity} ) | | 102.784 | | |
| #2 | | TITLE-ABS-KEY ( "physical activit*" ) | | 202.572 | | |
| #3 | | INDEXTERMS ( "Exercise" ) | | 379.807 | | |
| #4 | | INDEXTERMS ( {Exercise Therapy} ) | | 36.125 | | |
| #5 | | TITLE-ABS-KEY ( exercise ) | | 625.532 | | |
| **#6** | | #1 OR #2 OR #3 OR #4 OR #5 | | 786430 | | |
| #7 | | INDEXTERMS("counseling") | | 133.741 | | |
| #8 | | ( "referral"  AND  INDEXTERMS ( "consultation" ) ) | | 64.484 | | |
| #9 | | *none* | |  | | |
| #10 | | INDEXTERMS ( "prescriptions" ) | | 158.553 | | |
| #11 | | TITLE-ABS-KEY ( "prescription*" ) | | 245.386 | | |
| #12 | | TITLE-ABS-KEY ( "prescribed physical activit*" ) | | 84 | | |
| **#13** | | #7 OR #8 OR #10 OR #11 OR #12 | | 433.841 | | |
|  | | ( INDEXTERMS ( "counseling" ) ) OR ( ( "referral" AND INDEXTERMS ( "consultation" ) ) ) OR ( INDEXTERMS ( "prescriptions" ) ) OR ( TITLE-ABS-KEY ( "prescription*" ) ) OR ( TITLE-ABS-KEY ( "prescribed physical activit*" ) ) | |  | | |
| #14 | | INDEXTERMS ( {primary health care} ) | | 91.829 | | |
| #15 | | INDEXTERMS ( {health promotion} ) | | 108.526 | | |
| #16 | | TITLE-ABS-KEY ( "primary care physician*" ) | | 22020 | | |
| #17 | | TITLE-ABS-KEY ( "general practi*" ) | | 198.316 | | |
| #18 | | TITLE-ABS-KEY ( "primary care intervention*" ) | | 470 | | |
| **#19** | | #14 OR #15 or #16 OR #17 OR #18 | | 385.885 | | |
|  | | ( INDEXTERMS ( {primary health care} ) ) OR ( INDEXTERMS ( {health promotion} ) ) OR ( TITLE-ABS-KEY ( "primary care physician*" ) ) OR ( TITLE-ABS-KEY ( "general practi*" ) ) OR ( TITLE-ABS-KEY ( "primary care intervention*" ) ) | |  | | |
| **#20** | | #6 AND #13 AND '!) | | 3107 | | |
|  | | ( ( INDEXTERMS ( "Motor Activity" ) ) OR ( INDEXTERMS ( "physical activit*" ) ) OR ( INDEXTERMS ( "Exercise" ) ) OR ( TITLE-ABS-KEY ( exercise ) ) OR ( INDEXTERMS ( "Exercise Therapy" ) ) ) AND ( ( INDEXTERMS ( "counseling" ) ) OR ( ( "referral" AND INDEXTERMS ( "consultation" ) ) ) OR ( INDEXTERMS ( "prescriptions" ) ) OR ( TITLE-ABS-KEY ( "prescription*" ) ) OR ( TITLE-ABS-KEY ( "prescribed physical activit*" ) ) ) AND ( ( INDEXTERMS ( {primary health care} ) ) OR ( INDEXTERMS ( {health promotion} ) ) OR ( TITLE-ABS-KEY ( "primary care physician*" ) ) OR ( TITLE-ABS-KEY ( "general practi*" ) ) OR ( TITLE-ABS-KEY ( "primary care intervention*" ) ) ) | |  | | |
|  | | *after filters applied* | | **3065** | | |
|  | | **Remaining** | | **3429** | | |
|  | | **Updated on 31.01.2023** | | **419** | | |
| **Web of Science Core Collection** (21.06.2020; updated on 31.01.2023) | | | | | |  |
| **Nr.** | | | **Query string** | **Hits** | |  |
|  | | | ***Stage one*** *(12:30;13:40)* |  | |  |
| #1 | | | TOPIC: ("exercise referral scheme*") | 109 | |  |
| #2 | | | TOPIC: ("exercise referral") | 136 | |  |
| #3 | | | ALL FIELDS: ("exercise prescription scheme*") | 4 | |  |
| #4 | | | TOPIC: ("exercise on prescription") | 27 | |  |
| #5 | | | TOPIC: ("physical activity prescription") | 82 | |  |
| #6 | | | TOPIC: ("physical activity referral scheme*") | 8 | |  |
| #7 | | | TOPIC: ("green prescription") | 27 | |  |
| #8 | | | ALL FIELDS: (physical activity prescription scheme) | 66 | |  |
| #9 | | | TOPIC: ("physical activity referral") | 13 | |  |
| **#10** | | | #1 OR #2 OR #3 OR #4 OR #5 OR #6 OR #7 OR #8 OR #9 | 295 | |  |
|  | | | TOPIC: ("exercise referral scheme*") OR TOPIC: ("exercise referral") OR TOPIC: ("exercise prescription scheme*") OR TOPIC: ("exercise on prescription") OR TOPIC: ("physical activity prescription") OR TOPIC: ("physical activity referral scheme*") OR TOPIC: ("green prescription") OR TOPIC: (physical activity prescription scheme) OR TOPIC: ("physical activity referral")  Indexes=SCI-EXPANDED Timespan=1990-2020 |  | |  |
|  | | | **Remaining** | **295** | |  |
|  | | | ***Stage two*** |  | |  |
| #1 | | | TOPIC: ("motor activit*") | 14.582 | |  |
| #2 | | | TOPIC: ("physical activit*") | 140.574 | |  |
| #3 | | | TOPIC: (exercise) | 333.652 | |  |
| #4 | | | TOPIC: ("exercise therap*") | 3.833 | |  |
| #5 | | | *none because the translation is same as #3* |  | |  |
| **#6** | | | #1 OR #2 OR #3 OR #4 | 437220 | |  |
| #7 | | | TOPIC: (counsel$ing) | 69427 | |  |
| #8 | | | TOPIC: (referral AND consultation) | 4848 | |  |
| #9 | | | TOPIC: ("directive counsel$ing") OR TOPIC: ("Motivational Interviewing") | 3036 | |  |
| #10 | | | TOPIC: (prescription*) | 88037 | |  |
| #11 | | | *none because the translation is same as #10* |  | |  |
| #12 | | | TOPIC: (physical activit* NEAR/5 prescri*) | 576 | |  |
| **#13** | | | #7 OR #8 OR #10 OR #12 | 162778 | |  |
|  | | | TOPIC: (counsel$ing) OR TOPIC: (referral AND consultation) OR TOPIC: ("directive counsel$ing") OR TOPIC: ("Motivational Interviewing") OR TOPIC: (prescription*) OR TOPIC: (physical activit* NEAR/5 prescri*) |  | |  |
| #14 | | | TOPIC: ("primary health care") | 17051 | |  |
| #15 | | | TOPIC: ("health promotion") | 20638 | |  |
| #16 | | | TOPIC: (primary care physician*) | 40582 | |  |
| #17 | | | TOPIC: (general practi*) | 150855 | |  |
| #18 | | | TOPIC: (primary care NEAR intervention*) | 6463 | |  |
| **#19** | | | #14 OR #15 or #16 OR #17 OR #18 | 217357 | |  |
|  | | | TOPIC: ("primary health care") OR TOPIC: ("health promotion") OR TOPIC: (primary care physician*) OR TOPIC: (general practi*) OR TOPIC: (primary care NEAR intervention*) |  | |  |
| **#20** | | | #6 AND #13 AND #19 | 1634 | |  |
|  | | | TS=("motor activit*" OR "physical activit*" OR exercise OR "exercise therap*") AND TS=(counsel$ing OR (referral AND consultation) OR "directive counsel$ing" OR "Motivational Interviewing" OR prescription* OR physical activit* NEAR/5 prescri*) AND TS=("primary health care" OR "health promotion" OR primary care physician* OR general practi* OR primary care NEAR intervention*) |  | |  |
|  | | | *filter applied* | **1929** | |  |
|  | | | **Remaining** | **1929** | |  |
|  | | | **Search update on 31.01.2023** | **125** | |  |
| **NIHR-HTA** (stage 1: 22.06.2020, stage 2: 23.06.2020; both updated on 31.01.2023) | | | | | |  |
| **Nr.** | | | **Query string** | **Hits** | |  |
|  | | | ***Stage one*** |  | |  |
| #1 | | | (exercise referral scheme*) | 5 | |  |
| #2 | | | *none* |  | |  |
| #3 | | | (exercise prescription schemes) | 0 | |  |
| #4 | | | (exercise on prescription) | 2 | |  |
| #5 | | | (physical activity prescription) | 0 | |  |
| #6 | | | (physical activity referral scheme*) | 0 | |  |
| #7 | | | (green prescription) | 1 | |  |
| #8 | | | (physical activity prescription scheme) | 0 | |  |
| #9 | | | (physical activity referral) | 1 | |  |
| **#10** | | | #1 OR #4 OR #7 OR #9 | 9 | |  |
|  | | | (exercise referral scheme*) OR (exercise on prescription) OR ((green prescription) OR (physical activity referral)) | **9** | |  |
|  | | | ***Stage two*** |  | |  |
| #1 | | | MeSH DESCRIPTOR Motor Activity | 1334 | |  |
| #2 | | | (physical activity) | 895 | |  |
| #3 | | | MeSH DESCRIPTOR Exercise | 1137 | |  |
| #4 | | | MeSH DESCRIPTOR Exercise Therapy | 1055 | |  |
| #5 | | | (exercise) | 3457 | |  |
| **#6** | | | #1 OR #2 OR #3 OR #4 OR #5 | 4037 | |  |
| #7 | | | MeSH DESCRIPTOR Counseling | 490 | |  |
| #8 | | | MeSH DESCRIPTOR Referral and Consultation | 432 | |  |
| #9 | | | MeSH DESCRIPTOR Directive Counseling | 81 | |  |
| #10 | | | MeSH DESCRIPTOR Prescriptions | 170 | |  |
| #11 | | | (prescription) | 102 | |  |
| #12 | | | (referral) OR (prescribed physical activit*) | 187 | |  |
| **#13** | | | #7 OR #8 OR #9 OR #10 OR #11 OR #12 | 1280 | |  |
| #14 | | | MeSH DESCRIPTOR Primary Health Care | 1405 | |  |
| #15 | | | MeSH DESCRIPTOR Health Promotion | 889 | |  |
| #16 | | | MeSH DESCRIPTOR Physicians, Primary Care | 16 | |  |
| #17 | | | (general practi*) | 175 | |  |
| #18 | | | (primary care intervention*) | 8 | |  |
| **#19** | | | #14 OR #15 or #16 OR #17 OR #18 | 2387 | |  |
| **#20** | | | #6 AND #13 AND #19 | 9 | |  |
|  | | | **Remaining** | **18** | |  |
|  | | | **Search update on 31.01.2023** | **0** | |  |
| **CINAHL** (30.04.2021; updated on 31.01.2023) | | | | | |  |
| **Nr.** | **Query string** | | | | **Hits** |  |
|  | ***Stage one*** | | | |  |  |
| #1 | TI exercise referral scheme* OR AB exercise referral scheme* | | | | 99 |  |
| #2 | TI exercise referral OR AB exercise referral | | | | 283 |  |
| #3 | TX exercise prescription scheme* | | | | 13 |  |
| #4 | TI "exercise on prescription" OR AB "exercise on prescription" | | | | 91 |  |
| #5 | TI "physical activity prescription" OR AB "physical activity prescription" | | | | 63 |  |
| #6 | TI physical activity referral scheme* OR AB physical activity referral scheme* | | | | 30 |  |
| #7 | TI green prescription OR AB green prescription | | | | 36 |  |
| #8 | TX physical activity prescription scheme* | | | | 2 |  |
| #9 | TI physical activity referral OR AB physical activity referral | | | | 100 |  |
| **#10** | #1 OR #2 OR #3 OR #4 OR #5 OR #6 OR #7 OR #8 OR #9 | | | | **283** |  |
|  | ((((((((exercise referral scheme*[Title/Abstract]) OR (exercise referral[Title/Abstract])) OR (exercise prescription schemes)) OR (exercise on prescription[Title/Abstract])) OR (physical activity prescription[Title/Abstract])) OR (physical activity referral scheme*[Title/Abstract])) OR (green prescription[Title/Abstract])) OR (physical activity prescription scheme)) OR (physical activity referral[Title/Abstract]) | | | |  |  |
|  | ***Stage two*** | | | |  |  |
| #1 | (MM "Physical Activity") OR (MM "Exercise") | | | | 53.698 |  |
| #2 | TI ( physicial activity or exercise ) OR AB ( physicial activity or exercise) | | | | 118.921 |  |
| #3 | "exercise therapy" | | | | 17.205 |  |
| **#4** | #1 OR #2 OR #3 | | | | 53.698 |  |
| #5 | (MM "Referral and Consultation") | | | | 13.681 |  |
| #6 | "prescription" | | | | 56.234 |  |
| #7 | TI prescription OR AB prescription | | | | 43.845 |  |
| #8 | TI ( referral or referral process or referral pathway or care pathway) OR AB (referral or referral process or referral pathway or care pathway) | | | | 53.641 |  |
| #9 | TI prescribed physical activity OR AB prescribed physical activity | | | | 109 |  |
| **#10** | #5 OR #6 #7 OR #8 OR #9 | | | | 131.663 |  |
| #11 | (MM "Primary Health Care") | | | | 40.463 |  |
| #12 | (MM "Secondary Health Care") | | | | 368 |  |
| #13 | (MM "Health Promotion") | | | | 42.407 |  |
| #14 | (MM "Physicians") | | | | 31.558 |  |
| **#15** | #11 OR #12 OR #13 OR 14 | | | | 113.304 |  |
| #16 | #4 AND #10 AND #15 | | | | 207 |  |
|  | **Remaining** | | | | **439** |  |
|  | **Search update on 31.01.2023** | | | | **128** |  |
| **CORE** (26.06.2020; updated on 31.01.2023) | | | | | |  |
|  | ***Stage one*** | | | |  |  |
| #1 | title:("exercise referral scheme*") abstract:("exercise referral scheme*") | | | | 313 |  |
| #2 | title:("exercise referral") abstract:("exercise referral") | | | | 858 |  |
| #3 | "exercise prescription scheme*" | | | | 139 |  |
| #4 | title:("exercise on prescription") abstract:("exercise on prescription") | | | | 194 |  |
| #5 | title:("physical activity prescription") abstract:("physical activity prescription") | | | | 215 |  |
| #6 | title:("physical activity referral scheme*") abstract:("physical activity referral scheme*") | | | | 59 |  |
| #7 | title:("green prescription") abstract:("green prescription") | | | | 99 |  |
| #8 | "physical activity prescription scheme" | | | | 0 |  |
| #9 | title:("physical activity referral") abstract:("physical activity referral") | | | | 225 |  |
|  | **Total** | | | | **274** |  |
|  | **Search update on 31.01.2023** | | | | **36** |  |
| **Open grey** (30.06.2020; updated on 31.01.2023) | | | | | |  |
|  | ***Stage one*** | | | |  |  |
| #1 | exercise referral scheme* | | | | 9 |  |
| #2 | exercise referral | | | | 15 |  |
| #3 | exercise prescription schemes | | | | 1 |  |
| #4 | exercise on prescription | | | | 26 |  |
| #5 | physical activity prescription | | | | 19 |  |
| #6 | physical activity referral scheme* | | | | 4 |  |
| #7 | green prescription | | | | 4 |  |
| #8 | physical activity prescription scheme | | | | 1 |  |
| #9 | physical activity referral | | | | 5 |  |
| **#10** | "exercise referral scheme*" OR "exercise referral" OR "exercise prescription scheme*" OR "exercise on prescription" OR "physical activity prescription" OR "physical activity referral scheme*" OR "green prescription" OR "physical activity prescription scheme" OR "physical activity referral" | | | | 21 |  |
|  | exercise referral scheme* OR exercise referral OR exercise prescription scheme* OR exercise on prescription OR physical activity prescription OR physical activity referral scheme* OR green prescription OR physical activity prescription scheme OR physical activity referral | | | | **57** |  |
|  | **Search update on 31.01.2023** | | | | **14** |  |
| **Google scholar** (30.06.2020)  Weekly search alerts until 06.04.2022 | | | | | |  |
| **Nr.** | **Query string** | | | | **Records** |  |
|  | **Stage one** | | | |  |  |
| #1 | allintitle: exercise referral scheme | | | | 121 |  |
| #2 | allintitle: exercise referral | | | | 0 |  |
| #3 | "exercise prescription scheme*" | | | | 253 |  |
| #4 | allintitle: exercise on prescription | | | | 71 |  |
| #5 | allintitle: physical activity prescription | | | | 328 |  |
| #6 | allintitle: physical activity referral scheme | | | | 33 |  |
| #7 | allintitle: green prescription | | | | 76 |  |
| #8 | allintitle: physical activity prescription scheme | | | | 1 |  |
| #9 | "physical activity prescription scheme" | | | | 2 |  |
| #10 | allintitle: physical activity referral | | | | 125 |  |
| #11 | Total | | | | 1010 |  |
|  | without duplicates | | | | **771** |  |
|  | **Search alerts until 06.04.2022** | | | | **70** |  |
| **Journals** | | | | | |  |
|  | BMC Public Health | | | | **30** |  |
|  | European Journal of General Practice | | | | **2** |  |
|  | Scandinavian Journal of Public Health | | | | **7** |  |
|  | British Journal of Sports Medicine (BJSM) | | | | **14** |  |
|  | Scandinavian Journal of Medicine & Science in Sports | | | | **7** |  |
|  | Australian and New Zealand Journal of Public Health | | | | **60** |  |
| **Websites** | | | | | |  |
|  | National Institute for Health and Care Excellence (NICE) https://www.nice.org.uk/ | | | | **151** |  |

**Additional file 2.** Overview of included studies sorted by comparison group

| **Reference, country, model** | **Study design, name, period** | **Objective** | **Sample characteristics** | **Follow-up time** | **Intervention/**  **Comparator** | **Results** | **Author’s conclusions** |
| --- | --- | --- | --- | --- | --- | --- | --- |
|  |  |  |  |  |  |  |  |
| **PARS vs. usual care/written information** *(14 studies, 6780 participants)* | | | | | | | |
| Aittasalo et al. 2006 [39]  Finland,  Prex | RCT (physician as unit of randomization),  2003-2004 | To examine the effectiveness of prescription-based counseling and self-monitoring in the promotion of PA in primary health care. | Inactive, 85 % with chronic illness  n = 203 (154 women)  I = 130, C = 73  Age: 20-65 | 2, 6 months | **I:** (Prex)**:** 5-10 min prescription-based PA counseling based on the 5 A's framework, prescription  **C:** usual care | *Between-group analysis*  **Total PA min/week** (SR): + 118 and 79 min at 2 and 6 months°  **MVPA min/week** (SR): + 21 and 16 min at 2 and 6 months°, ND  **PA sessions/week** (SR): + 1 session (p = 0.05) at 2 months, at 6 months°  **MVPA PA sessions/week** (SR): + 0.8 sessions and 0.9 sessions at 2* and 6 months* favoring Prex | The Prex increased the weekly frequency of patients’ overall PA in short-term and at least moderate-intensity PA at 2 and 6 months. Prex can be recommended as a tool for primary health care physicians to promote PA. |
| Kallings et al. 2009b [41]  Sweden,  Physical Activity on Prescription (PAP) | RCT,  January-June 2006 | To evaluate the efficacy of PAP to reduce cardiometabolic risk factors in elderly women and men with low PA, overweight and abdominal obesity. | Inactive, overweight and abdominal obesity  n = 101 (43 women)  I = 47, C = 54  Age: 68-68 | 6 months | **I:** 30 min of individualized participant-centered counseling resulting in a written PAP  **C:** usual care + one page written information about PA importance for health | *Between-group analysis*  **MVPA min/week** (SR): +*  **MVPA sessions/week** (SR): +*  **+ ≥ 3000 steps/day** (OM): 32% vs. 14 %*  *Within-group analysis:*  **I**: +* | PAP has great potential to become an important method for promoting PA in older overweight women and men with a low PA level and abdominal obesity. |
| Morén et al. 2016 [58]  Sweden,  PAP | RCT,  June 2010-October 2013 | To objectively measure the effect of PAP on PA and physical capacity, as well as self-rated health at 3 and 6 months after TIA. | Inactive, Acute Transient Ischemic Attack  n = 88 (47 women)  I = 44, C = 44  Age: 49-90 | 3 (mid-scheme), 6 months (post-scheme) | **I:** Individualized participant-centered counseling resulting in a written PAP, follow-up at 3 & 6 months  **C:** usual care, oral and written information on stroke risk factors | *Between-group analysis*  **MVPA min/day** (OM): ND  **Steps/day** (OM): Tendency toward a difference | No significant differences between groups were found in PA at 3 and 6 months. |
| Lawton et al. 2008 [47]  New Zealand,  enhanced Green Prescription (GRx)  ([93]‡) | RCT,  2004-2005 | To assess the effectiveness of a primary care based program of exercise on prescription among relatively inactive women over a two-year period. | Inactive women  n = 1089  I = 544, C = 545  Age: 40-79, 58.9 ± 7 | 12 (3 months post-scheme), 24 months (15 months post-scheme) | **I:** 7-13 min brief advice led by nurse and written prescription, 30 min face-to-face follow-up at 6 months, monthly telephone support over 9 months (average of 5 calls, each lasting 15 min)  **C:** usual care | *Between-group analysis*  **Total PA min/week** (SR): +, median (IQR), 233 (43) vs 165 (30) at 12 months*; 214 (39) vs 179 (33) at 24 months*  **No. reaching PA guidelines** (SR): +, 43% vs 30% at 12 months*; 39% v 33% at 24 months*  *Within-group analysis:*  **I**: +*; **C**: +* | This program increased PA over two years, although falls and injuries also increased. |
| Samdal et al. 2019 [61]  Norway,  Healthy Life Centres (HLC) model | Pragmatic RCT,  June 2014-September 2015 | To evaluate the effect of behavior change interventions at Norwegian Healthy Life Centres on participants’ moderate to vigorous intensity PA six months after baseline. | Inactive, with or at risk of NCDs  n = 118 (91 women)  I = 57, C = 61  Age: 48.6 ± 13.4 | 6 months (3 months post-scheme) | **I:** referral to a 12 weeks group-based behavior change intervention, with an individual counseling session based on MI at entry and exit  **C:** usual care (wait listing) | *Between-group analysis*  **MVPA h/day** (OM): ND  I as reference, *b* (95% CI) for C, - 0.04 (-0.25, 0.18) | This RCT identified no effect on change in MVPA time after a six months’ intervention period. |
| Murphy et al. 2012 [46]  UK,  The Welsh National Exercise Referral  Scheme (NERS)  ([96, 97]‡) | Pragmatic RCT, recruitment July 2007-October 2008 | To assess the effectiveness and cost effectiveness in increasing PA and reducing anxiety and depression among participants referred for CHD risk and/or anxiety, depression and stress. | Inactive, CHD risk, mild to moderate depression, anxiety or stress  n = 2160 (1415 women)  I = 1080, C = 1080  Age: 16-88, mean 52 ± 14.7 | 12 months (post-scheme) | **I:** Three MI consultations at entry, 4-weeks and 16 weeks; access to one-to-one exercise instruction and/or 16-week tailored supervised group exercise program; 8-months telephone contact; 12-month review  **C.a:** usual care and a leaflet highlighting the benefits of exercise, addresses of local facilities  **C.b:** usual care (wait listing) | *Between-group analysis*  **Total PA min/week** (SR): + for all participants° (OR 1.19, 95% CI 0.99 to 1.43):  + for CHD only* (OR 1.29, 95% CI 1.04 to 1.60);  ND for mental health group or mental health and CHD group | NERS was effective in increasing PA among those referred for CHD risk only. Among mental health referrals, NERS did not influence PA. |
| Taylor et al. 1998 [63]  UK,  Exercise Referral Programme | RCT | To evaluate a fully operational GP referral scheme, in terms of modifying PA, blood pressure, smoking, and body composition over a nine-month period. | Inactive, at risk of CHD  N = 142 (89 women)  I = 97, C = 45  Age: 40-70 | 16 (6 weeks post-scheme), 26 (16 weeks post-scheme), 37 weeks (27 weeks post-scheme) | **I:** Prescription card (used for referral), initial, mid and final assessment, 10-week exercise program  **C:** usual care (wait listing) | *Between-group analysis*  **EE kcal/kg/day** (SR): ND  **Moderate PA min/week** (SR): +°  **Vigorous PA min/week** (SR): +* at 16 weeks, +° at 26 and 37 weeks | Referral to the exercise program led to largely short-term increases in PA. |
| Elley et al. 2003  [48]  New Zealand,  Green Prescription (GRx)  ([94, 95]‡) | Cluster RCT,  April 2000-2001 | To assess the long-term effectiveness of the “Green Prescription” program, a clinician based initiative in general practice that provides counseling on PA. | Inactive  n = 878 (582 women)  I = 451, C = 427  Age: 40-79 | 12 months (9 months post-scheme) | **I:** screening, advice (7-13 min) from primary care professional resulting in PA goals written on a prescription, telephone support (10-20 min) over 3 months; mailed materials; feedback at the next visit  **C:** usual care (wait listing) | *Between-group analysis*  **Total EE kcal/kg/week** (SR): +, mean changes (95% CI), 9.38 (3.96-14.81)*  **Leisure PA kcal/kg/week** (SR): +, 2.67 (0.48-4.86)*  **Leisure exercise min/week** (SR): +, 33.6 (2.4-64.2)* | GRx is effective in increasing PA over 12 months. |
| Gademan et al. 2012 [67]  The Netherlands,  Exercise on Prescription (EoP) | Controlled trial | To evaluate the effect of EoP in physical inactive women living in multi- ethnic deprived neighborhoods in the Netherlands. | Inactive women from ethnic minority groups, 90% overweight/obese  n = 514  I = 192, C = 322  Age: 16-85 | 6 (post-scheme), 12 months (6 months post-scheme) | **I:** referral to EoP, intake, 18 once a week supervised PA sessions including individual advice on PA, final evaluation, referral to Exercise without Prescription (exit route)  **C:** usual care | *Between-group analysis*  **Total PA MET-min/week** (SR): ND at 6 and 12 months  **Leisure PA MET-min/week** (SR): +*, mean diff (95% CI), 316 (43, 589) at 6 months*; 432 (158, 706) at 12 months* | EoP had a small positive effect on PA during leisure time (short-term and long-term) as well as on PA during household activity (long-term), but no effect was found on the total amount of PA. |
| James et al. 2017 [52]  Australia,  Referral to Accredited Exercise Physiologists  ([98]‡) | Pragmatic RCT (three arm), NewCOACH trial,  2011-2014 | To (1) determine the efficacy of primary care physicians’ referral of insufficiently active participants for counseling to increase PA, compared with usual care, and (2) compare the efficacy of face-to-face counseling with counseling predominantly via telephone. | Inactive, at least one chronic disease  n = 203 (143 women)  I = 132, C = 71  Age: 20-85, 57 ± 13 | 12 months (10 months post-scheme) | **I:** 60-minute initial consultation and four 30-minute follow-up appointments with an accredited exercise professional.  **C:** usual care + mailed health promotion brochure | **Steps/day** (OM): +*, mean diff 1,002 steps (95% CI: 244, -1759)* | Referral to expert PA counseling resulted in small but important improvements in activity that were maintained for 9 months after intervention completion. |
| Livingston et al. 2015 [56]  Australia,  ENGAGE | Cluster RCT, ENGAGE  October 2011-June 2013 | To determine the efficacy of a clinician referral and exercise program in improving  exercise levels and quality of life for men with prostate cancer. | Inactive, prostate cancer (majority stage I or II)  n = 147 (all men)  I = 54, C = 93  Age: 39-84, 65.6 ± 8.5 | 12 weeks (post-scheme) | **I:** referral to a 12-weeks supervised exercise program (comprising 2 gym sessions and 1 home-based session per week)  **C:** usual care with minimal information about PA | *Between-group analysis*  **MVPA:** No effect  min/week (SR): No effect, *d* (95% CI), 0.08 (-0.28 – 0.45)  min/day (OM): ND, 0.17 (-0.33 – 0.67)  **Vigorous PA:** Mixed results  min/week (SR): +*, 0.46 (0.09 – 0.82)  min/day (OM): -0.18 (-0.68 – 0.32)  **Moderate PA:** No effect  min/week (SR): -0.03 (-0.40 – 0.33)  min/day (OM): 0.33 (-0.17 – 0.83)  **Meeting PA guidelines** (OM): +*, 0.75 (0.35 – 0.12) | There was a significant effect on subjective measured vigorous PA. There were no significant intervention effects based on objectively measured PA. There was no effect on subjective measured MVPA or moderate PA. |
| Smith et al. 2000 [99]  Australia,  Active Practice | Controlled trial,  Active Practice | To investigate the impact of a simple written prescription for PA given by a general practitioner and the effect of supplementing this with mailed information materials about PA. | Inactive  n = 680 (445 women)  I.a = 238, I.b = 233,  C = 209  Age: 25-65 | 6-10 weeks, 7-8 months | **I.a:** prescription for exercise  **I.b:** prescription for exercise, a mailed stage matched booklet about two weeks after seeing their doctor  **C:** usual care | *Between-group analysis*  **Total PA min/week** (SR): small change but not significant  **No increasing PA by ≤ 60 min/week** (SR):  I.a vs C, +°, OR (95%CI), 1.59 (1.00-2.52) at 6-10 weeks; 1.19 (0.71-1.97) at 7-8 months  I.b vs C, +*, 1.63 (1.04-2.55) at 6-10 weeks, 1.78 (1.08-2.94) at 7-8 months | The prescription, when supplemented by a stage matched information booklet, was associated with modest short-term improvements. The prescription alone did not lead to significant improvements. |
| Martín-Borràs et al. 2018 [57],  Spain,  Exercise referral scheme (ERS) | RCT,  January-March 2009 | To assess the effectiveness of a primary care based ERS linked to municipal resources and enhancing social support and social participation in establishing adherence to PA among adults over a 15-month period. | Inactive, at least one NCD  n = 422 (257 women)  I = 220, C = 202  Age: 18-85 | 6 (3 months post-scheme), 12 months (9 months post-scheme), 15 months (1 year post-scheme) | **I:** 12-week PA program, linkage to community resources, social support and social participation  **C:** usual care, were called once every 4 weeks to minimize drop-outs (healthy lifestyle advices, reminder of the following assessment session) | *Between-group analysis*  **Total PA MET-min/week** (SR): +* at all follow-ups  **Vigorous PA MET-min/week** (SR): No difference  **Moderate PA MET-min/week** (SR): +* at all follow-ups  **Walking** **MET-min/week** (SR): +* at all follow-ups | The ERS is an effective and sustainable intervention in maintaining and increasing of self-reported PA levels in the long term. |
| Galaviz et al. 2013 [100]  Canada,  PA prescription + referral | 2 (pre–post) by 3 (treatment group) mixed design (physician as unit of randomization),  2010 | To compare the effectiveness of a PA prescription plus referral intervention versus a prescription only intervention delivered in primary care. | Inactive, BMI ≥ 35 kg/m^2^  n = 35 women  Age: 25-45 | Pre-post | **I.a:** PA counseling (< 3min), PA prescription and referral to a community program  **I.b:** PA counseling (< 3 min) and PA prescription  **C:** usual care | *Between-/-within-group analysis*  **Total PA min/week** (SR): +* in I.a and I.b, no effect on C group; I.a and I.b more effective than C group  **No meeting PA guidelines** (SR): I.a, 17% to 50%; I.b, 25% to 58%, no effect on C group; I.a and I.b more effective than C group | Both prescription interventions resulted in similar improvements in PA compared with usual care. |
| **PARS vs. Advice** *(5 studies, 1856 participants)* | | | | | | | |
| Bendrik et al. 2021 [40]  Sweden,  enhanced PAP | RCT,  2010-2015 | To evaluate whether PA on prescription, comprising five sessions, was more effective in increasing PA than an one-hour advice session after six months. | Inactive, knee/hip osteoarthritis  n = 141 (102 women)  I = 72, C = 69  Age: 40-74, 60.3 ± 8.30 | 6 months (post-scheme) | **I:** 60 min individualized participant-centered counseling, PA prescription, structured follow-up (15-60 min, at three weeks, 3 months and 6 months), group booster session on PA and osteoarthritis  **C:** 60 min general advice on osteoarthritis and PA  Both groups included behavior change techniques | *Between-group analysis*  **Total PA min/week** (SR): ND  **MVPA min/day** (OM): ND  **Total EE kcal/day** (OM): ND  **Steps/day** (OM): ND  *Within-group analysis*  **Total PA min/week** (SR):  **I**: +*, 105 (95% CI 75–120) to 165 (95% CI 135–218); **C**: +*, 75 (95% CI 75–105) to 150 (95% CI 120–225) | Individually tailored PAP and four follow- ups do not materially improve PA level more than individualized advice about osteoarthritis and PA. Patients increased self-reported PA but not accelerometer-assessed PA. |
| Gallegos-Carrillo et al. 2017 [49]  Mexico,  ERS  ([101, 102]‡) | pragmatic cluster RCT (primary healthcare centers as unit for randomization),  2011-2012 | To evaluate the effectiveness of providing brief behavioral PA counseling in primary care facilities, compared with an ERS among inactive or insufficiently active hypertensive adults. | Inactive, mild hypertension > 5 years  n = 232 (163 women)  I = 117, C = 115  Age: 35-70 | 16 weeks (post-scheme), 24 weeks (8 weeks post-scheme) | **I:** screening, brief PA counseling (15 min), referral, 16-week group-based exercise program (48 moderate-intensity, 1-hour group-based PA sessions, three sessions/week)  **C:** screening, brief PA counseling (15 min) | *Between-group analysis*  **MVPA min/week** (OM): ND  **No meeting PA guidelines**: ND  *Within-group analysis*  **MVPA min/week** (OM): **I**: + 40 min*; **C**: + 53 min*  **No meeting PA guidelines**: **I**: + 51.9%*; **C**: + 53.9%* | Both brief PA counseling and exercise referral led to modest improvements in PA levels, with no significant differences between groups. The impact was influenced by program attendance. |
| Isaacs et al. 2007 [51]  UK,  Fitness for Life, ‘Walking Partners’ scheme | RCT (3 arms), Exercise Evaluation Randomized Trial (EXERT),  October 1998-April 2002 | To evaluate and compare the effectiveness and cost-effectiveness of a leisure center-based exercise program, an instructor-led walking program and advice only in participants referred for exercise by their GPs. | Inactive, CVD risk  n = 943 (all referrals, 635 women)  I.a = 317  I.b = 311  C = 315  Age: 40-74, 57.04 ± 8.73 | 10 weeks (post-scheme), 6 months (4 months post-scheme), 1 year (10 months post-scheme; only I.a & I.b) | **I.a:** referral to a 10-week program of supervised exercise classes (2-3 times/week), two fitness assessments at baseline and after 10 weeks  **I.b:** referral to a 10-week community-based instructor-led walking program, ‘Walking Partners’, with over 20 walks in different parts of the borough (2-3 times/week)  **C:** tailored advice, information on PA & local exercise facilities (wait listing) | *Within/-between-group analysis*  **No meeting PA guidelines** (SR): +* in all groups at 10 weeks, 6 months & 1 year; ND  **Total PA min/week** (SR): +* in I.b only at 10 weeks, 6 months & 1 year; +17%* I.b vs C  **MVPA min/week** (SR): +* in all groups at 10 weeks & 6 months; +38%* I.b vs I.a; +53%* I.b vs C  **Total EE** (SR): +* in I.b only at 10 weeks, 6 months & 1 year  **Adherence**: higher in the l.a than I.b (X^2^ = 63.9, 4 df, p <0.001) | The inclusion of supervised exercise classes or walks as a formal component of the scheme may not be more effective than the provision of information about their availability. |
| Pfeiffer et al. 2001 [59]  USA,  GRx  replication study of Swinburn 1998 [62] | RCT, Ohio study,  February-August 1999 | To determine if a written exercise prescription increases PA when added to verbal advice. | Inactive  n = 49 (44 women)  I = 24, C = 25  Age: 62-92, 74 ± 1.1 | 6 weeks | **I:** verbal advice, PA prescription  **C:** verbal advice | *Between-group analysis*  ND in any of the outcomes (Total PA, leisure and walking)  *Within-group analysis*  **Total PA min/week** (SR): I, +116 min*; C, +180 min*  **Leisure PA min/week** (SR): I, +24 min; C, +38 min | The current study does not support the greater effectiveness of verbal plus written exercise advice over verbal advice alone in increasing PA among older adults. |
| Swinburn et al. 1998 [62]  New Zealand,  GRx | RCT,  May-August 1995 | To assess the impact of written vs verbal advice from a general practitioner on PA over 6 weeks in sedentary individuals. | Inactive  n = 491 (281 women)  I = 239, C = 252  Age: 49 ± 15 | 6 weeks | **I:** verbal advice, PA prescription  **C:** verbal advice | *Between-group analysis*  **Nr participating in leisure PA** (SR): +* in favor of I  **PA participation** (SR): +* in favor of I | The Green Prescription was more effective than verbal advice alone in increasing the PA level over a 6-week period. |
| **PARS vs. prescription/referral** *(3 studies, 890 participants)* | | | | | | | |
| Galaviz et al. 2013 [100]  Canada,  PA prescription + referral | See above | See above | See above | See above | See above | See above | See above |
| Riera-Sampol et al. 2020 [60]  Spain,  Majorca model | RCT,  March 2015-January 2018 | To evaluate the effectiveness of a multifactorial intervention in increasing adherence to PA prescription in participants with two or more cardiovascular risk factors. | Inactive, at least 2 CVD risk factors  n = 310 (153 women)  I = 150, C = 160  Age: 35-75, 62.2 ± 8.8 | 12 months (post-scheme) | **I:** 1:1 face-to-face MI resulting in PA prescription, PA assets booklet, follow-up visit at 2, 6 and 9 months, stage-based content  **C:** general PA prescription | *Between-group analysis*  **Adherence to PA recommendations**: +* in favor of I (χ2 = 3.951, p = .047)  **Total PA MET-h/week** (SR): ND  **Walking MET-h/week** (SR): + in favor of I but not significant | The multifactorial intervention performed by primary care nurses induced a higher adherence to the 150-min of weekly PA recommendation. |
| Harrison et al. 2005a [50]  UK,  ERS | RCT,  March 2000-December 2001 | To assess the effectiveness of a primary care referral scheme on increasing PA at 1 year from referral. | Inactive, CHD risk factors  n = 545 (363 women)  I = 275, C = 270  Age: 18-60+ | 6 months (3 months post-scheme), 9 months (6 months post-scheme), 12 months (9 months post-scheme) | **I:** referral, tailored 1-h consultation, subsidized 12 week leisure pass, encouraged to attend at least two center-based sessions a week, information about non-leisure center based activities available across the Borough, exit consultation  **C:** referral form, written information by post, information about non-leisure center based activities available across the Borough | *Between-group analysis*  **No participating ≥ 90 MVPA min/week** (SR): ND at 12 months (5.4%) (OR, 95% CI; 1.49, 0.86-2.57, p=0.16), 9%* at 6 months (OR, 95% CI; 1.67,1.08-2.60, p=0.05) | Community-based PARS have some impact on reducing sedentary behavior in the short-term, but which is unlikely to be sustained and lead to benefits in terms of health. |
| **Enhanced support/high dose vs. standard/low-dose PARS** *(11 studies, 2961 participants)* | | | | | | | |
| Buckley et al. 2020 [73]  UK,  Coproduced PA referral scheme (Co-PARS) | Three-arm quasi­experimental trial | To investigate the effectiveness of Co­PARS compared with a usual care ERS and no treatment for increasing cardiorespiratory fitness. | Inactive, NCD(s)  n = 68 (25 women)  I = 33, C.a = 19, C.b = 16  Age: ≥ 18 | 12 weeks (mid-scheme), 6 months (10 weeks post-scheme) | **I:** 12-week subsidized access to a fitness center as usual care ERS, 60 min induction, followed by 30 min one-to-one behavior change consultations at weeks 4, 8, 12 and 18  **C.a:** usual ERS, 12­week subsidized access to a fitness center, initial 1-hour induction, 12-week exercise program  **C.b:** lifestyle advice booklet | *Between-group analysis (*I vs C.a, I vs C.b)  **Light PA min/day** (OM): ND  **Moderate PA min/day** (OM): ND  **Vigorous PA min/day** (OM): ND | No significant changes in PA at 12 weeks or 6 months were noted. |
| Lundqvist et al. 2020 [42]  Sweden,  enhanced PAP  ([90, 103]‡) | RCT,  2010-2014 | To explore possible differences between the PAP interventions concerning PA-level and other outcomes. | Inactive after a 6-months PAP  n = 190 (94 women)  I = 98, C = 92  Age: 27-77 | 1 (mid-scheme), 2 years (post-scheme) | **I:** standard Swedish PAP, plus enhanced support: additional 9 follow-ups throughout 2 years, aerobic physical fitness test, ergometric bicycle  **C:** standard Swedish PAP | *Between-/-within-group analysis*  **PA level** (SR): I, +62.9%*; C, +50.8%*; ND  **Total PA MET-min/week** (SR): I, +*;C, +*; ND | This study demonstrated that during continued, long-term, 2-year intervention with two PAP treatment strategies, the PA level increased, with no significant differences between groups. |
| Fortier et al. 2011 [55]  Canada,  Intensive Physical Activity Counselling (IPAC) | RCT, Physical Activity Counselling (PAC) trial,  May-September 2005 | To evaluate the incremental value of intensive PA counseling by an onsite exercise professional (PA counselor) over and above that of brief counseling by a physician. | Inactive, NCD(s) (diabetes, arthritis, CVD, hypertension, depression/anxiety, other)  n = 120 (83 women)  I = 61, C = 59  Age: 18-69, 47.3 ± 11.1 | 6 weeks (mid-scheme), 13 (post-scheme),19 & 25 weeks (3 months post-scheme) | **I:** brief (2-4 min) PA counseling, 6 additional participant-centered counseling sessions over 3 months, PA prescription  **C:** brief (2-4 min) PA counseling, PA prescription | *Between-/-within-group analysis*  **LTPA level** (SR): I, +* at 6 weeks, effect size=0.81; C, +* at 6 weeks, effect size=0.17; +* group difference in favor of I at 6 & 13 weeks, ND at 19 weeks & 25 weeks (12 weeks post-I)  **Activity counts/min, moderate/vigorous PA min/day** (OM): ND | Multiple face-to-face patient-centered PA counseling sessions lead to short-term PA increases only when assessed by questionnaire. No group differences were found up to 12 weeks post intervention. |
| Taylor et al. 2020 [64]  UK,  ERS + e-coachER  ([104, 105]‡) | Pragmatic RCT, September 2016-  April 2017 | To determine if adding the e-coachER intervention to ERS is more clinically effective and cost-effective in increasing PA after 1 year than usual ERS. | Inactive, at least one NCD  n = 420 (152 women); 232 analyzed  I = 224, C = 226  Age: 18-75, 51.1 ± 13.2 | 4, 12 months (post-scheme) | **I:** web-based behavioral support (pedometer, fridge magnet, PA recoding sheets, user guide for the web-based support), referral to usual ERS (3 different ERS)  **C:** usual ERS (3 different ERS) | *Between-group analysis*  **MVPA min/week in ≥ 10-minute bouts**  (OM): ITT complete-case comparison at 12 months, n=232, mean difference (95%CI), 11.8 (-2.1-26), p=0.10  ND for any of the secondary outcomes at 12 months.  *Between-group analysis*  **MVPA min/week in ≥ 10-minute bouts**  (OM): I, +° at 4 & 12 months; C, +* at 4 months, -° at 12 months  **Uptake**: no effect, I=75%, C=78% | Offering e-coachER support had only small but non-significant effects on objectively recorded MVPA compared with usual ERS at 12 months. |
| Petrella et al. 2010 [65]  Canada,  Step Test Exercise Prescription Stage of change counseling (STEPS) | Cluster RCT (family physician practices as randomization unit), August 2000-January  2002 | To determine the effects of adding stages of change–based counseling to an exercise prescription for older, sedentary adults in family practice. | Inactive  n = 360 (206 women)  I = 193, C = 167  Age: 55-85, 64.9 ± 7.1 | 12 months (post-scheme) | **I:** individualized exercise prescriptions based on submaximal step test results, stage-matched advice  **C:** prescriptions based on submaximal step test results | *Between-group analysis*  **Total EE Kcal/kg/day** (SR): +* in favor of I: mean change (95%CI), 0.42(0.12-0.72)  *Within-group analysis*  **Total EE Kcal/kg/day** (SR): I, +*; mean change (95%CI), 0.67(0.43-0.91); C, +*, 0.25(0.07-0.42); | Addition of staged behavioral support to the exercise prescription appeared to promote better long-term behavior. |
| Romé et al. 2009 [43]  Sweden,  enhanced PAP  ([91]‡) | RCT,  February 2006-December 2007 | To analyze costs and consequences of changing physical activity behavior due to the “Physical Activity on Prescription” (PAP) program. | Inactive  n = 525 (358 women)  I = 268, C = 257  Age: 20-80, 51.9 ± 13.5 | 4 months (post-scheme), 12 months (8 months post-scheme) | **I:** standard PAP, referral to local sport clubs for supervised exercise sessions (45-60 min 2/week, instructed to exercise on their own once a week), 2 hours PA education, individual motivational counseling (physiotherapists).  **C:** standard PAP, written information about the possibility to participate in supervised exercise 1/week on a moderately intense level | *Within-/between-group analysis*  **Total PA MET-min/week** (SR): In both groups more than 2x increase*; ND, Mann–Whitney U-test=0.936, p=0.681  **Self-perceived active PA level**: 65% of I, 53% of C | Exercise twice a week, motivational counseling, and education compared with exercise only once a week does not make any difference in improving PA. |
| Sørensen et al. 2008 [44]  Denmark,  Exercise on Prescription (EoP) | RCT,  2005-2006 | To compare the short- (0–4 months) and long-term (0–10 months) effects of high-intensive EoP intervention (counseling and supervised exercise) with a low-intensive intervention (counseling). | Inactive  n = 52 (31 women)  I = 28, C = 24  Age: 53.4 (95%CI, 49.8-57.1) | 4 months (post-exercise intervention), 10 months (post-scheme) | **I:** 5 health profiles (questions about lifestyle and health) and motivational counseling sessions (45-60 min) at baseline, 2, 4, 7 and 10 months, 4-month group-based training intervention (24 1-h sessions in total)  **C:** 3 health profiles (questions about lifestyle and health) and motivational counseling sessions (45-60 min) at baseline, 4 and 10 months | *Between-group analysis*  **Total PA min/week** (SR): delta (95%CI), 48(-31,128) at 4 months; 10(-74,94) at 10 months  *Within-group analysis*  **Total PA min/week** (SR):  I, delta (95%CI), 63* (13,114) at 4 months, 20(-34,73) at 10 months  C, delta (95%CI), 23 (-0.47,0.02) at 4 months, 20(-45,85) at 10 months | No added value of the high-intensive EoP intervention compared with the low-intensive intervention was found. |
| Bredahl et al. 2011 [85]  Denmark,  EoP  ([106]‡) | Quasi-experimental study,  2005-2009 | To explore the long-term effect of prescribed exercise on levels of self-efficacy, stage of change and PA. | Inactive  n = 337 (231 women  Age: 54.5 (12.9) | 4 (post-exercise intervention), 10 (post-scheme), 16 months (6 months post-scheme) | **I:** referral to a 4-month group-based training intervention (24 1-h sessions in total), counseling sessions (45-60 min) at baseline and 4 months, voluntary phone based and/or personal motivational counseling after 10 and 16 months.  **C:** counseling sessions (45-60 min) at baseline and 4 months, voluntary phone based and/or personal motivational counseling after 10 and 16 months | *Between-group analysis*  **PA** (SR): ND  *Within-group analysis*  **PA** (SR): +* | EoP improves participants’ levels of PA and stages of change, regardless of the intensity of the intervention (counseling versus counseling and exercise). |
| Duda et al. 2014 [45]  UK,  Birmingham Exercise on Prescription scheme | Cluster RCT(leisure centers as unit of randomization), November 2007-July 2008 | To examine within arm change and compare the effect of an ERS based on Self-Determination Theory with a standard ERS on participants’ self-reported physical activity and other health outcomes. | Inactive, medically controlled  n = 347 (253 women)  I = 184, C = 163  Age: <30-65+ | 3 months (post-scheme), 6 months (3 months post-scheme) | **I:** referral, 1-h initial PA consultation, fitness assessment, self-management exercise promotion booklet, follow-up by telephone or in person at 1 (15-20 min) and 2 months (5 min), exit 20-30 min consultation at 3 months and again the option for a fitness assessment  **C:** referral, 1-h initial PA consultation, fitness assessment, 10-12 weeks exercise program, exit consultation | *Between-group analysis*  **MVPA min/week** (SR): mean diff., -.03, p=.93 at 6 months  *Within-group analysis*  **MVPA min/week** (SR):  I, mean diff. (95%CI), 196*(144-248) at 3 months, 114*(70-140) at 6 months  C, mean diff. (95%CI), 187*(131-243) at 3 months, 120*(67-172) at 6 months | Both standard provision and an SDT-based exercise referral program impacted self-reported PA levels. No difference between the SDT-based ERS and standard provision ERS was observed. |
| Andersen et al. 2020 [70]  Sweden,  PAP with counselor support | Observational follow-up study,  June 2013-June 2014 | To investigate differences in PA levels and HRQoL one year after PAP among participants who used physical counselor support in addition to PAP and participants who did not accept this offer of support and only received PAP. | Inactive  n = 400 (276 women)  I = 149, C = 251  Age: 18-90, 62 ± 14 | 12 months | **I:** PAP (short advice 5-10 min, individualized prescription), use of PA counselor support (Frequency of sessions based on the individual need of support)  **C:** PAP only group (did not use the counselor support) | *Between-group analysis*  **Total weekly PA** (SR): +* in favor of I, *r* = 0.259  **Exercise training** (SR): +* in favor of I, *r* = 0.260  **Everyday activity** (SR): +* in favor of I, *r* = 0.126  *Within-group analysis*  **Weekly PA** (SR): I, median diff (IQR), 2.0(7.0)*; C, 0.0(4.0)* | Patients who used PA counselor support attained a higher level of weekly PA one year after receiving PAP than patients who did not use counselor support. |
| Kolt et al. 2012 [53]  New Zealand,  Pedometer step-based GRx  ([107]‡) | RCT, Healthy Steps Trial,  July 2006-December 2007 | To compare the effectiveness of a pedometer-based Green Prescription with time-based (standard) Green Prescription. | Inactive  n = 330 (178 women)  I = 165, C = 165  Age: ≥ 65 | 3 (post-scheme), 12 months (9 months post-scheme) | **I:** Advice, step-based goal setting, 3 telephone counseling sessions by trained PA counselors over 3 to 4 months **C:** same but time-based instead of step-based goal-setting | *Between-group analysis*  **Total leisure PA min/week** (SR): ND, p=0.12  **Moderate PA min/week** (SR): ND, p=0.16  **Leisure walking min/week** (SR): I vs C, +63 min vs 30.9 min at 3 months; 49.6 min vs 28.1 min at 12 months* | Pedometer use resulted in a greater increase in leisure walking without any impact on overall activity level. |
| **Other comparison** *(4 studies, 3699 participants)* | | | | | | | |
| Foley et al. 2011 [92]  New Zealand,  Green Prescription (GRx) | Comparative study, 2007 | To compare the cost and outcomes of two  modes of delivery (community support and phone support) of a GRx program. | Inactive  n = 5441 referrals  I = 1562, C = 164  Age: 59 ± 13.8, 59.8 ± 14.24 | Pre-post | **I:** green prescription, weekly face-to-face support group meetings in which PA was offered in community setting (3-4 months)  **C:** Traditional green prescription, support involving monthly telephone calls over a 3–4-month period | **Nr of days meeting PA guidelines**: I vs C, 3.65 vs 2.66 days (95% CI: 0.30-1.70)*  **Uptake**: 31.9% (1735/5441)  **Adherence**: I, 8.5% (133/1572); C, 24.4% (45/163) completed the GRx programme | The two modes were comparable in cost and outcomes, though there was  greater penetration of target ethnic populations in community support. |
| Williams et al. 2017 [54]  New Zealand,  GRx extended version | Open-label randomized trial,  November 2008-February 2010 | To compare the effect of face-to-face and telephone modes of delivery of the national GRx program on participation and health outcomes on Māori and Europeans newly diagnosed with type-2 diabetes. | Inactive, type 2 diabetes  n = 138 (86 women)  I = 70, C = 68  Age: 30-86 | 6 months (post-scheme) | **I:** green prescription, monthly one-on-one face-to-face support for six months  **C:** green prescription, monthly telephone support for six months | **Uptake**: 100% (138/138) | The first interaction in GRx delivery should be face-to-face to improve uptake to participate (particularly among Māori), but the subsequent delivery can involve either face-to-face or telephone approaches. |
| Shepich et al. 2007 [66]  USA,  Exercise prescription | RCT | To investigate whether partial versus full subsidization and self- versus other monitoring promote adherence to physician-prescribed exercise. | Inactive, health conditions  n = 132 (88 women)  Age: 35-65 | 12 weeks (post-scheme) | **I:** screening, referral, initial assessment (health risk, fitness), individualized prescription: 12-weeks exercise program  **C:** self-monitoring vs. third party monitoring  full vs. partial subsidization | **Adherence:**  Partial subsidization& self-monitoring group: 11.55 ± 10.98 sessions  Partial subsidization& other monitoring group: 21.79 ± 13.75  Full subsidization& self-monitoring: 20.36 ± 14.37  Full subsidization & other monitoring: 22.45 ± 13.25 | Full subsidization and third-party monitoring increased exercise rates. These findings encourage use of both prescriptions to enhance prescribed exercise rates. |
| Hesketh et al. 2021 [88]  UK, Active Lifestyle ERS | Pragmatic trial,  Oktober 2017-March 2019 | To evaluate whether home-based high-intensity interval training added to an ERS improves cardiorespiratory fitness and adherence more than the traditional ERS. | Inactive, health conditions  N = 154 (66 women)  Age: 48 ± 10 | 12 weeks (post-scheme), 24 weeks (12 weeks post-scheme) | **I.a:** Traditional ERS (referral, initial consultation, exercise prescription, mid-consultation, final consultation)  **I.b:** ERS where instead of the traditional exercise prescription, a home-based HIIT was prescribed | **Uptake:**  59/67 for I.a, 60/87 for I.b  **Adherence** (weekly, %):  48 ± 35 for I.a, 39 ± 36 for I.b,ND | Adherence to both home-HIIT and traditional prescription was poor and similar for both groups. |
| **No comparison** *(21 studies, 28539 participants)* | | | | | | | |
| Kallings et al. 2008 [35]  Sweden,  Physical activity on Prescription (PAP) | Uncontrolled clinical trial,  2001-2003 | To evaluate the feasibility and effects of physical activity on prescription (FaR) in a routine clinical setting on PA level and quality of life after 6 months. | Inactive  n = 481 (361 women)  Age: 50.6 ± 13.4 | 6 months | **I:** Participant-centered physical PA counseling, individualized prescription, community-based network | **PA in daily life** (SR): +* at 6 months  **Exercise** (SR): +* at 6 months | Physical activity on prescription may be suitable as a conventional treatment in an ordinary primary health care setting to promote a more physically active lifestyle. |
| Kallings et al. 2009a [36]  Sweden,  Physical activity on prescription (PAP)  *sub-sample of Kallings et al. 2008* [35], *included separately for adherence* | Uncontrolled clinical trial,  2001-2003 | To examine self-reported  adherence to individualized prescribed PA in a routine primary health care setting. | Inactive  n = 240 (180 women)  Age: 51 ± 13 | 6 months | See [35] | **Adherence**: 65% adhered to PAP, 19% partly adhered, 16% nonadherence | At the 6-month follow-up a majority (65%) of the participants reported adherence to the prescription. |
| Leijon et al. 2009 [37]  Sweden, The Östergötland PA referral scheme (PAR) | Prospective cohort study,  2004-2005 | To analyze the effectiveness of a PAR scheme implemented in routine primary health care. | Inactive  n = 6300 (4221 women)  Age: 54 ± 14.6 | 3, 12 months | **I:** Participant-centered physical PA counseling, individualized prescription, follow-up phone call after 5 weeks only for those choosing faciltiy-based activities | **PA in daily life** (SR): +* at 3 and 6 months | PAR was successful in increasing self-reported PA among ordinary participants in routine PHC. |
| Leijon et al. 2010 [38]  [Leijon et al 2011 [89]]  Sweden, The Östergötland PAR  *same study as* [37], *included separately for adherence* | Prospective cohort study, 2004 | To assess the effectiveness of a Swedish PAR scheme by evaluating participants' self-reported adherence to PARs at 3 and 12 months. | Inactive  n = 3300 (1740 women)  Age: 54 ± 14.2 | 3, 12 months | See [37] | **Adherence**: 56% at 3 months, 50% at 12 months | Prescription from ordinary primary care staff yielded adherence in half of the participants in this PAR scheme follow-up. |
| Sjöling et al. 2011 [71]  Sweden,  PAP | Pre-post intervention with repeated measures,  2006-2007 | To investigate whether a combination of MI and PAP would increase leisure exercise time and subsequently improve health-related variables. | Inactive, mild to moderate hypertension  n = 31 (11 women)  Age: 43-71, 61.6 ± 7.0 | 3, 9, 15 months (post-scheme) | **I:** individualized prescription, four MI counseling sessions with a nurse at baseline, 3, 9, and 15 months | **Leisure exercise min/week** (SR): mean ± SD, baseline < 60 min/week, 195 ± 96* at 3 months, 251 ± 149* at 9 months, 300 ± 165* at 15 months | A 15 months intervention period with MI, in combination with PAP, significantly increased leisure exercise time. |
| Sørensen et al. 2011 [84]  Denmark,  EoP | Observational follow-up study,  2005-2009 | To analyze changes in PA and health-related quality of life among participants in five similar ‘Exercise on Prescription’ (EoP) programs. | Inactive  n = 449 (264 women)  Age: 52.7 ± 11.4 | 4 (post-exercise intervention), 10 (post-scheme), 16 months (6 months) | **I:** prescription, motivational counseling sessions and health profile assessments (four to five times within a 10-month period), supervised group-based training (24 sessions in total) | **Total PA MET-h/day** (SR): mean ± SD, + 0.64 ± 3.10* at 4 months, + 0.48 ± 2.80* at 10 months, + 0.27 ± 2.53* at 16 months (imputation: baseline carried forward) | Exercise on prescription can contribute to improvements in PA level in physically inactive participants with or at increased  risk of developing lifestyle diseases. |
| Dodd-Reynolds et al. 2020 [75]  UK, Northumberland ERS | Embedded mixed methods study,  June 2009-March 2014 | To explore equality of ERS experience, by examining the impact on weight status and PA, specifically accounting for social determinants of health, profession of referrer, and obesity grade. | Inactive, overweight or obesity  n = 3600 (2487 women)  Age: 51.3 ± 15.6 | 24 weeks (post-scheme), 52 weeks (6 months) | **I:** referral to local authority leisure sites, group-based supervised tailored exercise sessions (2/week, 48 sessions), motivational consultation pre-scheme, mid-scheme and post-scheme (24 weeks) | **Leisure exercise** (SR): mean (95%CI) PA score, + 16.299 (12.205-20.392)* at 24 weeks, + 4.325(-1.054-9.703) at 52 weeks | This study demonstrated success in increased PA and moderate weight loss for weight-loss referrals. |
| Hanson et al. 2013 [76]  UK, Northumberland ERS | Naturalistic observational cohort study,  July 2009-September 2010 | To investigate whether engagement in scheme consultations (1) resulted in increased PA and (2) was predicted by factors relating to the referral. | Inactive, NCD(s)  n = 2233 (1327 women)  Age: 53 ± 15.9 | 24 weeks (post-scheme) | **I:** referral to local authority leisure sites, group-based supervised tailored exercise sessions (2/week, 48 sessions), motivational consultation pre-scheme, mid-scheme and post-scheme (24 weeks) | **Leisure exercise** (SR): Participants who completed significantly increased their self-reported physical activity levels (t(638)= −11.55)* | Completers of the Northumberland ERS increased physical activity at 24 weeks, although the levels achieved were below the current UK guidelines of 150 min of moderate exercise per week. |
| Hanson et al. 2021 [77]  UK,  Scottish PARS | Longitudinal mixed methods study,  March 2018-January 2019 | To examine gender differences in uptake and adherence to a Scottish PARS and explore gendered perceptions of PARS experience. | Inactive, NCD(s)  n = 136 (65 women)  Age: 67 (IQR,55.0-73.7) | 12, 52 weeks | **I:** assessment of functionality level, referral to leisure and community centers, functionally stratified group PA sessions | **Total PA MET-min/week** (SR): +* only for female adherers and completers at 12 weeks but not maintained at 52 weeks; +* for non-starters at 52 weeks | We report short-term increases in PA for females resulting from PARS participation and significantly better uptake and adherence for those living in less deprived areas. |
| Pardo et al. 2014 [86]  Spain,  PAFES PARS | Longitudinal study,  2010-2011 | (1) To assess PA adherence after a six-month PARS, and at 12-months, and (2) to assess the impact of PARS on health-related quality of life in participants with cardiovascular risk factors | Inactive, at least two CVD risk factors  n = 323 (239 women)  Age: 62.6 ± 8.5 | 6 months (post-scheme), 12 months (6 months post-scheme) | **I:** referral to a 6-month supervised exercise program (3 sessions/week of 60 minutes of moderate PA) | **Total PA min/week** (SR): +* (n = 242, Wilcoxon test = 2.2*) post-scheme; ; -* at 6 months  **Moderate PA min/week** (SR): +* (n = 242, Wilcoxon test = 5.8*) post-scheme; -* at 6 months  **Vigorous PA min/week** (SR): - post-scheme and 6 months  **Walking** (SR): no change post-scheme and at 6 months | A six months PARS significantly increased PA level in participants with CVRF. Adherence to PA decreased at 12-month assessment. |
| Prior et al. 2019 [78]  UK,  ERS Tameside | Longitudinal follow-up study,  July 2015-2017 | To evaluate the long-term impact of participation in an ERS on self-reported PA and a range of health-related outcomes. | Inactive, at least one chronic condition or significant risk factors  n = 273 (153 women)  Age: 57.7 ± 14.1 | 24 (post-scheme), 52 weeks (6 months) | **I:** initial consultation, tailored PA opportunities program (options for supervised group-based activities or independent activities), follow-up consultations with the exercise professional at weeks 6, 12, and 24 weeks, 12 months of subsidized access to leisure facilities and supervised classes | **Total PA MET-min/week** (SR): +660* post-scheme, +636* from baseline to follow-up | Referral to a 6-month ERS led to significant improvements in PA and a range of health-related outcomes, which were sustained in the long term beyond the scheme’s duration. |
| Rödjer et al. 2016 [72]  Sweden,  PAP | Observational study, March 2007-February 2008 | To study the self-reported level of PA and quality of life in participants receiving physical activity on prescription for up to 24 months. | Inactive, NCD affected by PA  n = 146 (109 women)  Age: 55 ± 11 | 6, 12, 24 months | **I:** written individualized prescription, referred to a PAP coordinator, typically a physiotherapist, who conducted a participant-centered interview covering goal setting, level of motivation, regular support and follow-up.  Reference group: unmatched group of patients seeking primary healthcare (n = 58) | **MVPA** (SR): +* at 6 and 12 months, +° at 24 months (PA did not change in the reference group) | Participants receiving PAP showed an increased level of self-reported PA at six and 12 months. However, an observed positive change did not reach significance at 24 months. |
| Stewart et al. 2017 [79]  UK,  Active Living for Life (ALL) | Longitudinal repeated measures design,  April 2013-October 2015, | To investigate the effects of an exercise referral scheme aligned to the UK best practice guidelines. | Inactive, low or risk condition  n = 407 (243 women)  Age: 17 - 75+ | 6 weeks (mid-scheme), 12 weeks (post-scheme) | **I:** ERS designed following NICE guidelines | **Leisure PA** (SR): effect size pre-post, 1.32 for man and 1.15 for women | Participants increased their PA levels over the 12-week period, but the greatest improvements were obtained in the first 6 weeks. |
| Ward et al. 2010 [80]  UK,  Heartlinks | Before and after study,  2001-2007 | To identify changes in health, CHD risk and levels of PA amongst patients referred to a tailored exercise referral program. | Inactive, at risk of CHD  n = 279 (187 women)  Age: 24-88 | 12 months (post-scheme) | **I:** referral to a sports scientist (project officer), baseline assessment, general lifestyle advice, printed materials, consultations at 1, 3, 6 & 12 months | **MVPA sessions/week** (SR): Mean Difference, 1.789 (95% CI: 1.65 - 1.92), Range 0 – 6 sessions; 547% improvement (t = 13.06)* | The Heartlinks exercise referral model significantly increased PA levels over a 12 months period. |
| Webb et al. 2016 [68]  UK, The Welsh National  NERS - exercise program only | Cohort study (study 3) | To evaluate the impact of a NERS-delivered exercise-referral program with regard to the range of biomolecular, vascular hemodynamic, clinical and anthropometric parameters (study 3). | Inactive, pre-diabetic, obese  n = 14 (7 women)  Age: 56 ± 3.9 | 8 weeks (mid-scheme) | **I:** referral to a supervised 16-week group-based exercise program (35 minutes sessions 2/week) | **Total PA MET-min/week** (SR): 98 ± 142° vs baseline  **Steps/week** (OM): 3486 ± 469* vs. baseline | Participants underwent significant increases in PA levels. |
| Crone et al. 2008 [81]  UK,  Proactive scheme  ([108]‡) | Observational study, May 2000-May 2003 | To compare initial progression, uptake, and completion among participants referred on the basis of a mental health condition and those referred on the basis of physical health conditions. | Inactive, physical health or mental health referrals  n = 2901 (1636 women)  Age: 51 ± 14 (physical health referrals); 42 ± 14 (mental health) | Post-scheme | **I:** referral to PARS, 8-12 weeks of weekly/biweekly supervised exercise sessions | **Uptake**: 69% (1996/2901) for physical health referrals, 60% (79/134) for mental health referrals, p < 0.001  **Adherence**: 22% (29/134) mental health referrals, 34% (935/2767) for physical health referrals, p < 0.001 | PARS appears to be less well suited to the needs of the mental health patient. |
| Edmunds et al. 2007 [74],  UK,  Birmingham Exercise on Prescription scheme | Observational study | To examined differences in perceived autonomy support and other outcomes, between overweight/obese individuals who demonstrated greater adherence to an exercise on prescription program and those who adhered less. | Inactive, overweight/obesity  n = 49 (41 women)  Age: 44.98 ± 14.61 | 1, 3 months (post-scheme) | **I:** Initial consultation & assessment, fitness induction section; 12-weeks exercise plan followed independently at the leisure center but informal support/contact is maintained with the EoP advisor, final consultation with fitness assessment if appropriate, a report is sent to the GP | **Adherence**: 51% (25/49) still exercising at the end of EoP in accordance with their prescriptions, 4.1% were still exercising at the end of the 3-months but did not exercise in accordance with the amount of exercise prescribed | Exercise on prescription (EoP) schemes would benefit from creating services that foster self-determination via the facilitation of psychological need satisfaction. |
| Harrison et al. 2005b [69],  UK,  ERS | Prospective register-based study,  January 1998-December 2002 | To access the numbers and characteristics of participants referred and accessing a district-wide exercise referral scheme. | Inactive  n = 6610 (4016 women)  Age: 51.3 ± 12.6 | na | **I:** referral, tailored 1-h consultation (identification of a suitable PA program), subsidized 12 week leisure pass, encouraged to attend at least two center-based sessions a week, exit consultation | **Uptake**: 79% (5225/6610) | Primary-care participants seem to view the concept of exercise referral schemes positively but practitioners remain reluctant to refer many of their sedentary participants. |
| Lord et al. 1995 [82]  UK,  Stockport Exercise on Prescription Scheme | Pre-post design | To answer the two following questions regarding ERS: (1) ’do people turn up’?’ and (2) ’are people healthier having been prescribed exercise’?’ | Inactive  n = 419 (198 women)  Age: 18-65 | 10 weeks (post-scheme), 6 month (4 months post-scheme) | **I:** Referral, initial consultation, exercise program (1-h sessions 3/week) | **Uptake**: 60% (252/419)  **Adherence**: 30.5% (77/252) | A 60 percent initial uptake rate was  achieved. |
| Dinan et al. 2006 [83]  UK,  Exercise referral program | Prospective cohort  study | To assess the feasibility and effectiveness of a tailored exercise referral program for frail elderly patients delivered within a variety of inner city primary care settings. | Inactive, community dwelling frailer older  people  n = 242 | 8 weeks (post-scheme) | **I:** referral to a two-phase progressive exercise program (stage 1: 8-week exercise program in primary care; stage 2: transition to further exercise classes in community setting), telephone contact | **Uptake**: 87% (216/242)  **Adherence**: 82% (178/216) completed stage one, 52% (112/216) transferred to stage two | The program was effective in achieving participation in exercise sessions. |
| van de Vijver et al. 2022 [87]  The Netherlands, Referral to peer coach PA | Uncontrolled follow-up study | To evaluate the effects of linking a peer coach PA intervention to primary care through an exercise referral scheme. | Inactive older adults  n = 106  Age: ≥ 50 | 1 year (post-scheme) | **I:** referral to peer coach groups (1-h sessions on weekdays) | **Uptake:** 5.7% (6/106)  **Adherence:** 66.7% (4/6) | The effect of referral to the peer coach on was limited. |

*ERS* Exercise Referral Scheme, *n* sample size, *I* intervention, *C* control/comparison, *NCD* non-communicable disease, *MI* motivational interviewing, *SR* self-report, *OM* objective measure, *PA* physical activity, *MVPA* moderate to vigorous intensity PA, *EE* energy expenditure, *HIIT* High Intensity Interval Training, *+* increase or positive change, *CI* confidence intervals, *IQR* interquartile range, *** p < 0.05, *^°^* not significant, ND, no difference, MET metabolic equivalent of task, *d* Cohen’s *d*, *b* standardized regression coefficient, *CHD* coronary heart disease, *CVD* cardiovascular disease, *BMI* body mass index, *‡* report of the same study with no data extracted, *NA* Not Applicable

**Additional file 3**. Description of PARS components

| **Component** | **Description** |
| --- | --- |
| Person-centered approach | The study explicitly mentions that a patient-/person-centered approach to PA advice, counseling, or a PA plan was used. This approach places the participant at the center of the intervention by taking into account their past experiences, current activity level and motivation, capabilities, personal preferences, current needs and goals, interests, and beliefs about PA. The aim is to create a behavior change by promoting an interaction between the healthcare/exercise provider and the PARS participant, with shared decision-making and a respectful, empowering environment. Given that motivational interviewing is a person-centered method of communication and intervention delivery, studies that used this method were also counted as having this component. |
| Individualized content | The *content* of any PA advice, prescription, plan, or intervention is tailored/personalized to the individual’s heath status, needs, goals, abilities, and preferences (personalized content). Though similar to the person-centered approach, the aim in this case is to select appropriate intervention content that is health and behavioral change promoting.  International Classification of Health Interventions (ICHI) definition: “Working with the person to develop an individual plan including setting client goals and priorities, and identifying interventions, responsibilities and supports needed (services and resources) to achieve the goals” [PZB.TB.ZZ Individualized planning] |
| Behavior change theory | The PARS content design is based on a behavior change theory. |
| Behavior change techniques | The use of at least one behavior change technique. |
| Screening | Formal selection of participants that fit the scheme eligibility criteria (e.g., assessment of PA level in the waiting room or a screening questionnaire).  ICHI definition: “Using a questionnaire, rating scale or other instrument to test behavior concerning patterns of physical activity” [VEB.AC.ZZ Test of physical activity behaviours] |
| Brief advice | Advice from a healthcare professional about PA recommendations, health benefits, or other relevant information directly before issuing a prescription or referral form. The general aim is to bring up the PA topic in order to recommend that the participant take up the offer implied by a PA prescription or a referral to a PARS intervention.  ICHI definition: “Providing advice to encourage a change of behavior in relation to physical activity” [VEB.PN.ZZ Advising about physical activity behaviors] |
| Written materials | The participant is provided with printed materials, which can consist of educational information, motivational support, or a step-by-step guide to behavior change. |
| Written prescription | The participant receives a prescription, similar to a medical prescription, in which where individualized/standard PA recommendations or other PA opportunities are written down.  ICHI definition: “Instruction, direction or authoritative recommendation to obtain or pursue a specified health intervention, targeting patterns of behavior in relation to physical activity” [VEB.TI.ZZ Prescription for physical activity behaviors] |
| Referral | A standardized form used to refer the participant to another healthcare/exercise professional or exercise program. The referral form usually contains relevant medical information, the referral reason, and a contact for the participant to use in proceeding with the referral program. |
| Baseline consultation | A consultation session (30–60 minutes) before beginning with the PARS program, which may include an initial biopsychosocial assessment of the participant in order to create a PA plan or motivational counseling. Studies that perform only a baseline fitness assessment are also rated as having this component. |
| Exit consultation | A re-visit consultation (30–60 minutes) at the end of the scheme, with the purpose of progress evaluation or motivational support to continue PA after scheme completion. Studies that perform only an exit fitness assessment are also rated as having this component. |
| Counseling support session(s) | Counseling sessions (30–60 minutes) during the PARS program, with the aim of providing motivational support to help the participant in achieving their PA goals. It may involve the use of behavior change techniques, such as barrier management, relapse prevention, and feedback on behavior, or communication methods, such as motivation interviewing techniques, to influence theory-based constructs, such as self-efficacy, which ultimately results in behavior change.  ICHI definition: “Providing therapeutic and/or supportive communication in relation to patterns of behavior in relation to physical activity” [VEB.PP.ZZ Counselling about physical activity behaviors] |
| PA sessions | Structured, usually supervised, PA programs that are formal element of the scheme and are delivered by healthcare or exercise professionals. Studies that offer only subsidized access to local leisure facilities are not rated as including PA sessions.  ICHI definition: “Teaching, enhancing or developing skills - regarding behavior concerning patterns of physical activities - through practice” [VEB.PH.ZZ Training to influence physical activity behaviors; Training focused on execution of physical activities or exercises]; Organizing events or activities in which people can participate to support improved behavior concerning patterns of physical activity [VEB.RF.ZZ Providing opportunities for participation in relation to physical activity behaviors] |
| Education session(s) | Separate session (30–60 minutes) that focuses on education in PA.  ICHI definition: “Providing information to improve knowledge in order to influence behavior concerning patterns of physical activity” [VEB.PM.ZZ Education to influence physical activity behaviors] |
| Action for non-attendance | Any action undertaken if the participant did not attend PA or counseling sessions, with the aim of encouraging further PARS participation. |
| Structured follow-up | There is a the scheduled practice of proactively making contact with the participant in order to monitor adherence to the prescription or PA program. The follow-up is mostly made by phone and is shorter (5–20 min) and less intensive than a counseling support session. It may include feedback on progress, encouragement, relapse prevention, or making the necessary adjustments to the prescription or PA plan. |
| PA network | Explicit collaboration within the healthcare system (referrer professional, physiotherapist, and nurses) or outside the healthcare system (exercise professionals and PA providers), with the aim of creating a support network and PA opportunities for the participant during PARS the journey and after completion.  ICHI definition: “Working together and cooperating with relevant stakeholders to support improved behaviors concerning patterns of physical activity” [VEB.TD.ZZ Collaborating or building partnerships in relation to physical activity behaviors] |
| Feedback to referrer | The referring professional receives progress reports during or at scheme completion. |
| Exit strategies/routes | Strategies used to encourage participation in PA or signposting of participants to other PA opportunities after discharge from the scheme. This may involve including unassisted exercise sessions during the exercise program, explicit strategies to support PA independence after the scheme, making a self-directed PA plan, or a formal transfer to another program at scheme completion. |

*PA* physical activity, *ICHI* International Classification of Health Interventions

**Additional file 4.** Overview of PARS models characteristics sorted by country

| **PARS model** | **Year introduced and implemented** | **PARS length** | **Theoretical underpinning, delivery approaches** | **Behavior change**  **techniques** | **Referral/**  **prescription source, referral professional** | **Reason for referral** | **Referred to** | **Prescribed activities** | **PA sessions** | **Setting for prescribed activities/ scheme delivery** |
| --- | --- | --- | --- | --- | --- | --- | --- | --- | --- | --- |
| ***Finland*** | | | | | | | | | | |
| Prescription-based PA counseling (Prex) [39] | Developed and piloted during 2001-2002 | Open-ended | 5 As framework, patient-centered approach | Information about health consequences, goal setting, action planning, barrier management, self-monitoring | Primary healthcare, physician | Physical inactivity, no perceived obstacles for PA | Optional: physiotherapists, nurses, exercise experts | Emphasis on lifestyle activities | NA | Independent |
| ***Sweden*** | | | | | | | | | | |
| Physical Activity on prescription (PAP) [41] | 2010 | Open-ended | Social cognitive theory, transtheoretical model, motivational interviewing, supportive environment | Goal setting, action plan, information about health consequences | Primary healthcare, physician | Physical inactivity, overweight or abdominal obesity | Self-monitored or refered to public PA organizations | Lifestyle activities and/or structured exercise (individualized in regards to type of activity, frequency, duration) | NA | Independent or in public PA organizations |
| PAP [58] |  | 6 months | None noted, participant-centered approach | Goal setting | Secondary healthcare, physiotherapist | Physical inactivity, acute Transient Ischemic Attack | Self-monitored | Individualized lifestyle activities (walking, swimming) | NA | Independent |
| Enhanced PAP [40] | 2010 | 6 months | None noted, participant-centered approach | Information about health consequences, goal-setting (outcome goals), goal-setting (behavioral goal), action-planning, self-monitoring of behavior, review of behavior goals, graded tasks | Primary healthcare, physician, physiotherapist, nurse | Physical inactivity, hip/knee osteoarthritis | The referrer professional delivered the whole scheme | Lifestyle activities and/or structured exercise (individualized in regards to type of activity, frequency, duration) | NA | Independent or in PA organizations |
| PAP enhanced [42]  ([90, 103]‡) | 2020 | 2 years | Social cognitive theory, transtheoretical model, participant-centered approach, motivational interviewing | Goal setting, social support | Primary healthcare, practice nurse, physiotherapist | Physical inactivity after participating in a 6-months PAP, at least one component of metabolic syndrome | Physiotherapist | Lifestyle activities and/or structured exercise (individualized in regards to type of activity, frequency, duration) | NA | Independent or in PA organizations |
| PAP with counselor support [70] | In use since 2009 | 1 year | None noted, person-centered approach, motivational interviewing | None noted | Licensed health care professionals (e.g., physicians, nurses, physiotherapists) | Physical inactivity | PA counselor (licensed healthcare professionals, e.g., nurses or physiotherapists) | Lifestyle activities and/or structured exercise (individualized in regards to type of activity, frequency, duration) | NA | Independent or in local sport clubs |
| PAP enhanced [43]  ([91]‡) |  | 4 months | Transtheoretical model | Information about health consequences | Primary healthcare, physician, nurse, physiotherapist, occupational therapist, welfare officer, nutritionist | Physical inactivity, at least one chronic condition | Local sport clubs | NA | Two supervised exercise sessions (45 to 60 minutes) in local sport clubs twice a week; instructed to exercise additionally once a week on their own | Local sport clubs |
| PAP [35, 36] | 2001 | Open-ended | Social cognitive theory, transtheoretical model, motivational interviewing, supportive environment, patient-centered approach | Goal setting, self-monitoring of behavior | Primary healthcare, occupational health care, physiotherapist, medical doctor, nurse, other healthcare professionals | Physical inactivity, with or without lifestyle diseases | Self-monitored | Self-monitored or organized by public PA organizations  (Aerobic fitness training including walking, muscle strength training, mobility training, individual physical activity, group activity) | NA | Independent or public PA organizations |
| The Östergötland PA referral scheme  (PAR) [37, 38]  ([89]‡) | 2001 | Open-ended | Participant-centered approach | None noted | Primary healthcare, physician, nurse, physiotherapist, other healthcare professional | Physical inactivity, with or without lifestyle diseases (e.g. high blood pressure, diabetes, musculoskeletal, pulmonary disease) | Self-monitored or if the activity prescribed was facility based, a copy of prescription was sent to the PAR coordinator in the selected PA organization, who then contacted the participant by telephone or letter | Lifestyle activity (free-living or home-based activities such as walking) or a structured facility-based activity provided by a local PA organization or a combination of both | NA | Independent or public PA organizations |
| PAP [71] |  | 15 months | Motivational interviewing, patient-centered approach | Goal setting, self-monitoring of behavior, review of goals, discussion of barriers | Primary healthcare, family doctor | Physical inactivity, mild to moderate hypertension | Study nurse | Individual activities or group activities | NA | Independent or public PA organizations |
| PAP [72] |  | Open-ended | Participant-centered approach | Goal setting | Primary healthcare, physician, nurse, physiotherapist, dietician, etc. | Physical inactivity, condition that can be affected by PA (musculoskeletal, metabolic disorders, psychiatric disorders, other) | PAP coordinator (typically a physiotherapist) | Lifestyle activities or activities arranged by a public PA organization | NA | Independent or public PA organizations |
| **Norway** | | | | | | | | | | |
| Healthy Lifestyle Centers (HLC) model [61] |  | 12 weeks  (may be extended up to one year) | Self-determination theory, motivational interviewing | Information about health consequences, goal setting, PA plan, review goals, feedback on behavior | Primary healthcare, GP, other public service personnel,  or self-referral | Physical inactivity, existing chronic condition or at high risk | Healthy Life Centers (physiotherapists, trained lifestyle counsellors, PA instructors) | NA | 12-week group-based behavior change intervention (aerobic training, such as Nordic walking, light strength training, stretching and games) | primary care: Healthy Life Centers |
| **New Zealand** | | | | | | | | | | |
| Green Prescription (GRx) [48]  ([94, 95]‡) |  | 3 months | None noted, motivational interviewing | Goal setting | Primary healthcare, GP or practice nurse | Physical inactivity | A copy of the GRx was send to local sports foundation, where exercise specialists conducted the telephone support calls. | Individualized: usually home-based activities or walking | NA | Independent (usually home-based) |
| Enhanced GRx [47]  ([93]‡) |  | 9 months | None noted, motivational interviewing | Goal setting, action planning, barrier management, information about health consequences | Primary healthcare, practice nurse | Physical inactivity | community based regional sport trust (exercise specialist) | Moderate intensity PA such as brisk walking, with a goal of achieving 30 minutes five days a week | NA | Independent |
| GRx [62] |  | Open-ended | None noted | Goal setting | Primary healthcare, GP | Physical inactivity | Self-monitored | Individualized lifestyle activities |  | Independent |
| Pedometer step-based Green Prescription (GRx) [53]  ([07]‡) |  | 3 to 4 months | Transtheoretical model, motivational interviewing | Goal setting, information provision, review of goal(s), feedback on behavior, relapse prevention, self-monitoring of behavior | Primary healthcare, physician | Physical inactivity, elderly | Self-monitored | Lifestyle activities (e.g., walking rather than driving to the shopping center, participating in an older adult dance activity at the local community center) | NA | Home based or local facilities (dependent on the individuals goals) |
| GRx [92] |  | 3-4 months | None noted | Goal setting, action planning, social support, barrier management | Primary healthcare, GP, practice nurse | Physical inactivity | GRx patient support personnel | Lifestyle activities | NA | Independent or in community |
| GRx [54] |  | 6 months | Motivational interviewing | Goal setting | Primary healthcare, primary healthcare provider, GP, practice nurse | Physical inactivity, type 2 diabetes | Regional sports trust | The physical activity plans incorporated walking, swimming, weight training and utilized common activities such as washing clothes, vacuuming or gardening. | NA | Setting agreed to by the participant (most cases home based) |
| **USA** | | | | | | | | | | |
| GRx [59]  same scheme as Swinburn 1998 [62] |  | Open-ended | None noted | Goal setting | Primary healthcare, geriatrician | Physical inactivity, elderly | Self-monitored | Individualized lifestyle activities | NA | Independent |
| Exercise prescription [66] |  | 12 weeks | None noted | Monitoring of behavior by others/self-monitoring | Primary healthcare, physician | Physical inactivity, health condition | Exercise specialist | 12-week prescribed program including aerobic and strength training exercises | NA | Local community facility |
| **UK** | | | | | | | | | | |
| The Welsh National Exercise Referral  Scheme (NERS) [46]  ([96, 97]‡) | 2007 | 12 months (16-week exercise program) | None noted, motivational interviewing | Goal setting, monitoring, relapse prevention, review behavior goals | Primary healthcare, healthcare professionals | CHD risk, mild to moderate depression, anxiety or stress | Qualified exercise professionals | NA | One-to-one exercise instruction and/or group exercise classes | Local authority leisure centers |
| Exercise Referral Programme [63] |  | 10 weeks | None noted | None noted | Primary healthcare, GP | Physical inactivity, CHD risk (smoking, hypertension, overweight) | Leisure center (Hailsham Lagoon Leisure Centre, East Sussex) | NA | 10-week exercise program (moderate and vigorous aerobic type activity on various exercise machines, in a semi-supervised, informal environment) | Leisure center |
| Coproduced PA referral scheme (Co-PARS) [73] | 2017 | 18 weeks | Self-determination theory, motivational interviewing | Provide information, action planning, barrier identification/problem solving,  prompt self-monitoring of behavior, prompt review of behavioral goals, relapse prevention, coping planning | Primary healthcare, GP, physiotherapist, exercise physiologist, nurse, etc. | Physical inactivity, cardiometabolic, cancer, mental health, musculoskeletal | Exercise referral practitioner | Participants can choose a combination of gym, class and external physical activities to suit their preferences. Focus on integration of PA into individual lifestyles. | NA | Local authority fitness centers |
| Fitness for Life [51] | March 1995 | 10 weeks | [Unclear, evaluation measures included constructs of transtheoretical model, but it is not stated weather the scheme was based on theory] | None noted | Primary healthcare (sometimes from secondary healthcare), GP, practice nurse | Physical inactivity, CVD risk | Leisure center: fitness instructor | NA | Exercise classes: aerobics, body conditioning, aqua-aerobics, gymnasium and an optional swimming class | Leisure centers |
| ‘Walking Partners’ scheme [51] | NR | 10 weeks | None noted | None noted | Primary and secondary healthcare, GP, practice nurse, other healthcare professionals | Physical inactivity, CVD risk | Walking instructor | NA | Walking group program | 12 different locations around the borough parks |
| Exercise Referral Scheme [50, 69] | In place since 1997 | 12 weeks | None noted | None noted | Primary healthcare, primary care practitioner | Physical inactivity, CHD risk | Exercise officer | Encouraged to attend at least two center-based sessions a week, non-leisure center based activities |  | Council-run PA facilities across the Borough, leisure center |
| ERS + e-coachER [64]  ([104, 105]‡) | NR | 12 months | Self-determination theory | Self-monitoring of behavior, information about health consequences, information about emotional consequences, social support (practical), social support (emotional), social support (unspecified), goal-setting (behavior), action-planning, review behavior goals, restructuring the physical environment, avoidance/reducing exposure to cues for behavior, prompts/cues, reduce negative emotions | Primary healthcare (possible also from secondary healthcare), primary care practitioners (mostly by a GP, occasionally by a nurse) | Physical inactivity, one medical condition (hypertension, prediabetes, type 2 diabetes, lower limb osteoarthritis or a current/recent history of treatment for depression) | There are three different ERS providers that offer different programs;  The intervention group receives an additional web-based behavioral support designed to augment the scheme | NA | Depends on the ERS provider | Depends on the ERS provider |
| Birmingham Exercise on Prescription scheme (EoP) [45] | NR | 3 months | Self-determination theory, motivational interviewing (careful listening, parroting/ paraphrasing, handling resistance, and double-sided reflection) | Benefits and risks  of increased PA, goal setting, problem solving, barriers to PA, resources, social support, action planning | Primary healthcare, GP, practice nurse | Physical inactivity, motivation, at risk for or with chronic conditions | Health and fitness advisors (exercise professionals) | Individual and/or group activities | NA | Leisure center and in the community |
| Birmingham EoP [74] | NR | 12 weeks | Self-determination theory, motivational interviewing | Benefits and risks  of increased PA, goal setting, problem solving, barriers to PA, resources, social support, action planning | Primary healthcare, GP | Physical inactivity, CHD risk factors | Health and fitness advisors (exercise professionals) | 12-week prescribed exercise plan including activities on offer at the leisure center | NA | Leisure center |
| Northumberland Exercise Referral Scheme (ERS) [75] | NR | 24 weeks | Transtheoretical model | Potential barriers to increasing activity | Primary or secondary healthcare professional | Physical inactivity, overweight or obesity | Leisure provider | NA | Group-based sessions including gym, circuit classes, racquet sports and swimming, encouraged to undertake independent PA | Leisure facilities |
| Northumberland ERS [76] | NR | 24 weeks | Transtheoretical model | Potential barriers to increasing activity | Primary or secondary healthcare professional | Physical inactivity, chronic conditions | Leisure provider | NA | Group-based sessions including gym, circuit classes, racquet sports and swimming, encouraged to undertake independent PA | Leisure facilities |
| Scottish PARS [77] | NR | Open-ended | None noted | None noted | Primary or secondary healthcare professional | Physical inactivity, chronic conditions | 11 local authority leisure centers and 2 community facilities | NA | Functionally stratified group PA sessions. Activity options included once a week attendance at the gym, seated exercise classes, circuit classes or aqua-based activities. | Leisure centers, community facilities |
| ERS Tameside [78] | NR | 6 months | None noted | None noted (although stated that behavior change support was provided) | Health care professionals (including allied professions, e.g., physiotherapists, health trainers) | Physical inactivity, chronic conditions or at significant risk | Exercise professional | NA | Supervised group-based activities or independent activities, within the local leisure facilities (e.g., use of gym, swimming pool, and PA classes) or the community (e.g., health walks, walking football groups, home exercises) | Local leisure facilities, community or homebased |
| Active Living for Life (ALL) [79] | NR | 12 weeks | None noted (following NICE guidelines) | None noted | Health care professional | Physical inactivity, low or medium risk condition (CVD, weight management, respiratory, musculoskeletal, endocrine, neurological and mental health conditions) | ERS program/coordinator | Non-standardized, individualized mode of exercise focused predominantly on combination training | NA | Not reported |
| Heartlinks [80] | 2001 | 12 months | Transtheoretical model, motivational interviewing | Barrier management | Primary and secondary healthcare, all healthcare professionals (GP, practice nurse, pharmacists, occupational health nurses & "back to work" project staff, smoking cessation), self-referral | Physical inactivity, CHD risk | Project officer (sports scientist) | NA | Tailored activity options negotiated with the patient: home activity kits, Heartlinks class, guided walks, swimming, aquafit, gym, walking, slimswim, Tai Chi, independent home exercise, subsidized access to the local authority leisure facilities and to a local private health club, other | Home-based, outdoor, leisure facilities |
| NERS – exercise referral only [68] | NR | 16 weeks | None noted | None noted | Primary healthcare, GP | Physical inactivity, CVD risk (pre-diabetic, obese) | NERS exercise professional | NA | Exercise program comprised of twice-weekly 35 min group exercise sessions (walking-based gym sessions; NERS patients only) | Leisure centre |
| Proactive scheme [81] ([108]‡) | 1994 | 8 to 12 weeks | None noted | None noted | Primary and secondary healthcare, GP, practice nurse, physiotherapist, dietician, psychiatrist, nurse specialists, cardiac nurses, health visitors, smoking cessation officers, and healthy lifestyle coordinators | Physical inactivity, mental health or physical health problems | PARS co-ordinator (The co-ordinator assigned participants  to a leisure provider or were removed from PARS) | For those who don’t want to join the PARS formally, alternative activities such as home based activity or an increase in daily life activity, such as walking are offered. | 8 to 12 weeks of weekly/biweekly supervised exercise sessions (typically gym-based, but can include swimming, circuit training, or exercise-to-music classes, depending on participant preference and available facilities.) | Local leisure facilities |
| Stockport  Exercise on Prescription Scheme [82] | 1992 | 10 weeks | None noted | None noted | Primary healthcare, GP | Physical inactivity, CHD risk | Community health and fitness officer | NA | Exercise program: aqua-aerobics, cycling, tennis, weights, badminton, dancing, swimming, orienteering, yoga, bowls, keep fit, table tennis, walking, trampolining | Community leisure centers |
| Exercise referral program [83] | NR | 8 weeks | None noted | None noted | Primary healthcare, GP, nurse | Physical inactivity, frailer older people | Specialist exercise instructors | NA | Two-phase progressive exercise program: supervised chair-based strengthening exercises | Stage 1: primary care setting  Stage 2: leisure/community center |
| Active Lifestyle ERS [88] | NR | 12 weeks | None noted (in accordance with NICE guidelines) | Barrier management | Primary healthcare, GP | Physical inactivity | Lifestyle development officer | Moderate intensity continuous training 3x/week (gym equipment, exercise classes or exercise in the local environment) or home-HIIT 3x/week depending on preference | NA | Local gym or community, home |
| **The Netherlands** | | | | | | | | | | |
| EoP [67] |  | 6 months | None noted | Goal setting, coping strategies, social support | Primary healthcare, GP | Physical inactivity, ethnic minority | Lifestyle advisor, sport instructor | Daily activities like walking (Exercise without prescription, transition from the scheme) | 18 supervised PA sessions (Fitness, aquarobics, aerobics, dancing) | Training sessions were held in their neighborhood |
| Referral to peer coach PA [87] |  | 1 year | None noted | None noted | Primary healthcare, GP, practice nurse | Physical inactivity, adults ≥ 50 years old | Peer coach groups | NA | 1-h sessions on weekdays (focus on strength, flexibility,  coordination and stamina) | Outside, neighbourhood |
| **Denmark** | | | | | | | | | | |
| EoP [44] | 2002 | 10 months | Transtheoretical model, motivational interviewing | Barrier identification, goal setting | Primary healthcare, GP | Physical inactivity, motivation, with medically controlled disease (treatment scheme) or at risk (prevention scheme) | Physiotherapist | NA | 24 1-h supervised group-based training sessions (aerobic conditioning, e.g., Nordic walking and aerobics, light strength conditioning primarily using light weights and a high number of repetitions, stretching, and games) | Primary care |
| Exercise on Prescription (EoP) [84] | 2002 | 10 months | Transtheoretical model, motivational interviewing | Barrier identification, goal setting | Primary healthcare, GP | Physical inactivity, with or at risk of developing CVD, hypertension or type 2 diabetes (prevention scheme) | Physiotherapist | NA | Supervised group-based training sessions | Primary care |
| Exercise on Prescription (EoP) [85]  ([106]‡) | 2002 | 10 months | Transtheoretical model, motivational interviewing | Barrier management, goal setting, action planning | Primary healthcare, GP, self-referral | Physical inactivity, motivation, with medically controlled disease (treatment scheme) or at risk (prevention scheme) | Physiotherapist or exercise specialist | NA | Supervised group-based training (elements of aerobic exercise, e.g., Nordic Walking, Aerobic, strength training, stretching and games.) | Primary care |
| **Australia** | | | | | | | | | | |
| Referral to Accredited Exercise Physiologists [52]  ([98]‡) | 2006 | 13 weeks | Social cognitive theory, patient-centered approach | Information about health consequences of PA, goal setting [behavior], action planning, self-monitoring of behavior, discussion of barriers, problem solving, focus on past success, social support [unspecified], prompts or cues | Primary healthcare, physician | Physical inactivity, existing chronic condition | Accredited Exercise Physiologists (AEP) | Individualized | NA | Independent |
| ENGAGE [56] | 2011 | 12 weeks | Social cognitive theory | Goal setting, barrier management, social support | Secondary healthcare, clinicians working in urology and radiation oncology | Physical inactivity, prostate cancer survivor | Exercise physiologists | NA | 12-week exercise program (aerobic exercise, progressive resistance training, balance, and flexibility exercises) | Participant’s local community gym |
| Active Practice [99] | NR | Open-ended | Transtheoretical model | None noted | Primary healthcare, GP | Physical inactivity | Self-monitored | What doctors considered appropriate for each patient |  | Independent |
| **Spain** | | | | | | | | | | |
| ERS Catalonia [57] | NR | 12 weeks | None noted | Social support | Primary healthcare, healthcare professionals (GP, nurse) | Physical inactivity, existing chronic condition | PA specialist within primary care | NA | 12-week group-based PA program (aerobic activities, strength training), one session/week independent PA (brisk walking) | in a primary care available space or nearby outdoor spaces such as public parks |
| Majorca model [60] | NR | 12 months | Transtheoretical model, salutogenic model, motivational interviewing | Information about health consequences, pros and cons, goal setting, positive reinforcement, review outcome goal | Primary healthcare, primary care nurse | Physical inactivity, at least two CVD risk factors | The referrer professional delivered the whole scheme | Individualized | NA | Independent |
| PAFES PARS [86] | 2007 | 6 months | None noted | None noted | Primary healthcare, physician, nurse | Physical inactivity, at least two CVD risk factors | Supervised exercise program led by a PA professional | NA | Combination of moderate intensity activity, muscular endurance of the major muscle groups with use of equipment (dumbbells and elastic bands) and flexibility | Sports facility |
| **Mexico** | | | | | | | | | | |
| ERS Mexico [49]  ([101, 102]‡) | NR | 16 weeks | Social cognitive theory,  transtheoretical model | Nurses that delivered the brief counseling were trained in BCT, but it is not clear if one or more BCT were used. | Primary healthcare, physician, primary care nurse | Physical inactivity, mild hypertension > 5 years, contemplation/preparation phase | 16-week community-based exercise program delivered by fitness instructors | NA | 16-week PA exercise program (dancing, supervised walking, swimming) | Community-based sport facilities at Social Security Centers (primary healthcare) |
| **Canada** | | | | | | | | | | |
| PA prescription + referral [100] | 2010 | 8 weeks | 7 A`s framework, social cognitive theory | None noted | Primary healthcare, physician | Physical inactivity, overweight, obese women | Trained public health staff | 8 weekly 90-minute group educational sessions focusing on enhancing PA and healthy eating | NA | Local public health unit |
| Intensive Physical Activity Counseling (IPAC) [55] | NR | 3 months | Self-determination theory, social cognitive theory, motivational interviewing, 7 A’s framework | Goal setting, problem solving, social support, self-monitoring, review of goals, relapse prevention, barrier management, action planning | Primary healthcare, primary care provider (family physician, nurse practitioner) | Physical inactivity | PA counselor (within the same primary care center) | 1-month leisure-time PA goal, other tailored activities during intensive counseling | NA | Primary healthcare, independent |
| Step Test Exercise Prescription Stage of change counseling (STEPS) [65] | NR | Open-ended | Transtheoretical model | None noted | Primary healthcare, physician | Physical inactivity | No referral | Individualized based on submaximal step test results | NA | Independent |

*PA* physical activity, *ERS* Exercise Referral Scheme, *GP* general practitioner, *NICE* National Institute for Clinical Excellence, *HIIT* High Intensity Interval Training, NR Not Reported, *NA* Not Applicable, *‡* report of the same study with no data extracted

**Additional file 5.** PARS identified worldwide

| **Country** | **PARS model** | **Non included in the review**  **(reason)** | **Reference** |
| --- | --- | --- | --- |
| Australia | Newcastle Comparison Of Activity Coaching for Health (NewCOACH) |  | [52, 98] |
|  | ENGAGE |  | [56] |
|  | Active Practice |  | [99] |
| Belgium | Moving on Prescription [Bewegen Op Verwijzing (BOV)] | X (No outcomes, ineligible study design) | Smeyers et al. 2013 |
| Canada | PA prescription + referral |  | [100] |
|  | Intensive Physical Activity Counselling (IPAC) |  | [55] |
|  | Step Test Exercise Prescription Stage of change counseling (STEPS) |  | [65] |
|  | HealtheSteps™ | X (Provider perspectives) | Blunt et al. 2019 |
| Denmark | Exercise on Prescription (EoP) |  | [44, 84, 85, 106] |
| Finland | Prex |  | [39] |
| France | Physical activity on prescription – French law | X (Ineligible study design, language) | Poussel et al. 2018, Chenuel et al. 2020, Dranebois et al. 2021 |
| Germany | Physical Activity on Prescription [Rezept für Bewegung] | X (Ineligible study design) | Löllgen et al. 2013, Curbach et al. 2018, Emerich et al. 2020 |
|  | BewegtVersorgt | X (Ineligible study design) | Weissenfels et al. 2022 |
| Iceland | Physical activity on Prescription [Hreyfiseðill] | X (master and bachelor thesis) | Guðmundsdóttir 2015, Pálsdóttir 2016, |
| Ireland | National Exercise Referral Framework (NERF) | X (Ineligible study design/outcomes) | Woods et al. 2016 |
| Mexico | ERS Mexico |  | [49, 101, 102] |
| The Netherlands | Exercise on Prescription (EoP) |  | [67] |
|  | Care Sport Connectors (CSC) | X (Ineligible study design, providers perspectives) | Smit et al. 2015, Leenaars et al. 2016a, Leenaars et al. 2016b, Leenaars et al. 2017, Smit et al. 2020 |
|  | Referral to peer coach PA |  | [87] |
|  | BeweegKuur | X (Combined lifestyle intervention) | Berendsen et al. 2015 |
| New Zealand | Green Prescription (GRx) |  | [47, 48, 53, 54, 62, 92, 93, 94, 95, 107] |
| Northern Ireland | Healthwise Physical Activity Referral Scheme | X (Ineligible study design) | Belfast Health Development Unit 2013 |
| Norway | Healthy Lifestyle Centers (HLC) model |  | [61] |
| Peru | Prescription scheme | X (Ineligible setting, tertiary care) | Tenorio-Mucha et al. 2022 |
| Spain | ERS Catalonia |  | [57] |
|  | PAFES PARS |  | [86] |
|  | Majorca model (Multifactorial PA prescription intervention) |  | [60] |
| Sweden | PA on Prescription (PAP [FaR]) |  | [35–38, 40–43, 58, 70–72, 89–91, 103] |
| Switzerland | Physical Activity Promotion in Primary Care (PAPRICA) | X (No evaluation, ineligible study design) | Martin et al. 2014 |
| UK | The Welsh National Exercise Referral  Scheme (NERS) |  | [46, 96, 97, 68] |
|  | Exercise Referral Program [East  Sussex] |  | [63] |
|  | Coproduced PA referral scheme (co-PARS) |  | [73] |
|  | Fitness for Life (Barnet) |  | [51] |
|  | Walking Partners scheme |  | [51] |
|  | Exercise Referral Scheme |  | [50, 69] |
|  | Exercise Referral Scheme (ERS) + e-coachER |  | [64, 104, 105] |
|  | Birmingham Exercise on Prescription scheme |  | [45, 74] |
|  | Northumberland Exercise Referral Scheme |  | [75, 76] |
|  | Scottish PARS |  | [77] |
|  | ERS Tameside |  | [78] |
|  | Active Living for Life (ALL) |  | [79] |
|  | Heartlinks |  | [80] |
|  | Proactive scheme |  | [81, 108] |
|  | Stockport Exercise on Prescription Scheme |  | [82] |
|  | Exercise referral program |  | [83] |
|  | Scottish Borders General Practitioners Exercise Referral Scheme (GPERS) | X (Ineligible study design, postal survey) | Day and Nettleton 2001 |
|  | Let’s Get Moving | X (Ineligible study design) | Bull and Milton 2011 |
|  | South Gloucestershire Exercise on Prescription Scheme | X (Ineligible study design) | Flannery et al. 2014 |
|  | Glasgow General Practitioner Exercise Referral Scheme | X (Ineligible study design) | Greater Glasgow NHS Board 2002 |
|  | Various schemes in Scotland (n = 26) | X (Audit) | Buxton and McGeorge 2018 |
|  | Active Lifestyle ERS |  | [88] |
| USA | Green prescription |  | [59] |
|  | Exercise prescription (Wisconsin) |  | [66] |
| Global | Exercise is Medicine | X (Ineligible study design, training evaluation) | Lobelo et al. 2014, Fowles et al. 2018 |

Visit the online map for an overview of the PARS models: <https://maphub.net/emino/pars-models-worldwide>

# REFERENCES

Belfast Health Development Unit: Active Belfast. Healthwise Physical Activity Referral Scheme: SROI pilot exercise. Belfast; 2013.

Berendsen BAJ, Kremers SPJ, Savelberg HHCM, Schaper NC, Hendriks MRC. The implementation and sustainability of a combined lifestyle intervention in primary care: mixed method process evaluation. BMC Fam Pract. 2015;16:37. doi:10.1186/s12875-015-0254-5.

Blunt W, Gill DP, Riggin B, Brown JB, Petrella RJ. Process evaluation of the HealtheSteps™ lifestyle prescription program. Transl Behav Med. 2019;9:32–40. doi:10.1093/tbm/iby005.

Bull F, Milton K. Let's Get Moving: a systematic pathway for the promotion of physical activity in a primary care setting. Glob Health Promot. 2011;18:59–61. doi:10.1177/1757975910393173.

Buxton K, McGeorge S. Audit of Exercise Referral Schemes in Scotland: A snapshot of current practice. 2018. http://www.healthscotland.scot/media/2203/ers-review-scotland-final-report.pdf. Accessed 27 Apr 2020.

Chenuel B. Mise au point : la prescription médicale d’activité physique adaptée en pratique [The medical prescription for physical activity adapted in practice]. Nutrition Clinique et Métabolisme. 2020;34:116–21. doi:10.1016/j.nupar.2019.12.006.

Curbach J, Apfelbacher C, Knoll A, Herrmann S, Szagun B, Loss J. Physicians' perspectives on implementing the prevention scheme "Physical Activity on Prescription": Results of a survey in Bavaria. Z Evid Fortbild Qual Gesundhwes. 2018;131-132:66–72. doi:10.1016/j.zefq.2018.02.001.

Day F, Nettleton B. The Scottish Borders general practitioners exercise referral scheme (GPERS). Health Bull (Edinb). 2001;59:343–6.

Dranebois S, Lalanne-Mistrih ML, Nacher M, Thelusme L, Deungoue S, Demar M, et al. Prescription of Physical Activity by General Practitioners in Type 2

Diabetes: Practice and Barriers in French Guiana. Front Endocrinol (Lausanne). 2021;12:790326. doi:10.3389/fendo.2021.790326.

Emerich S, Preiser C, Rieger MA. Evaluation des Modellprojektes „Rezept für Bewegung“ in Baden-Württemberg mittels explorativer qualitativer Befragung. [Evaluation of the Model project "Prescription for Exercise" in Baden-Württemberg: An Explorative Qualitative Survey]. Gesundheitswesen 2020. doi:10.1055/a-1075-2149.

Flannery O, Loughren E, Baker C, Crone D. Exercise on prescription evaluation report for south Gloucestershire. Cheltenham; 2014.

Greater Glasgow NHS Board. Glasgow GP exercise referral scheme evaluation report. Glasgow; 2002.

Fowles JR, O'Brien MW, Solmundson K, Oh PI, Shields CA. Exercise is Medicine Canada physical activity counselling and exercise prescription training improves counselling, prescription, and referral practices among physicians across Canada. Appl Physiol Nutr Metab. 2018;43:535–9. doi:10.1139/apnm-2017-076

Guðmundsdóttir LÁ. Physical activity on prescription (PAP) as a resource of treatment in Iceland: General practitioners’ view: Bachelor thesis. 2015.

Leenaars KEF, Florisson AME, Smit E, Wagemakers A, Molleman GRM, Koelen MA. The connection between the primary care and the physical activity sector: professionals' perceptions. BMC Public Health. 2016b;16:1001. doi:10.1186/s12889-016-3665-x.

Leenaars KEF, Smit E, Wagemakers A, Molleman GRM, Koelen MA. Exploring the impact of the care sport connector in the Netherlands. BMC Public Health. 2017;17:813. doi:10.1186/s12889-017-4830-6.

Leenaars KEF, Smit E, Wagemakers A, Molleman GRM, Koelen MA. The role of the care sport connector in the Netherlands. Health Promot Int. 2016a;33:422–35. doi:10.1093/heapro/daw097.

Lobelo F, Stoutenberg M, Hutber A. The Exercise is Medicine Global Health Initiative: a 2014 update. Br J Sports Med. 2014;48:1627–33. doi:10.1136/bjsports-2013-093080.

Löllgen H, Wismach J, Kunstmann W. Das Rezept für Bewegung - Einsatzmöglichkeiten für Arzt und Patient: [Exercise prescription for health - Benefit for practitioner and patients]. Klinikarzt. 2013;42:416–20. doi:10.1055/s-0033-1358596.

Martin B, Padlina Oliver, Martin-Diener E, Bize R, Cornuz J, Kahlmeier S. Physical activity promotion in the health care setting in Switzerland. Schweizerische Zeitschrift für Sportmedizin und Sporttraumatologie. 2014;5:19–22. doi:10.1007/s12170-011-0180-6.

Pálsdóttir K. Physical activity on prescription (PAP): Study of efficiency and patients’ experience of the method: Master thesis. The University of Iceland. 2016.

Poussel M, Hupin D, Chenuel, Bruno, Edouard P. Physical Activity Prescription (PAP): The French model [Blog post]. BJSM. 2018.

Smeyers I, Deboutte F, Bartholomeeusen E. To move prescription : a handy tool to get patients moving. GP Now. 2013:238–41.

Smit E, Leenaars KEF, Wagemakers A, van der Velden K, Molleman GRM. Perceptions of Care Sport Connectors' Tasks for Strengthening the Connection Between Primary Care, Sports and Physical Activity: A Delphi Study. Int J Integr Care. 2020;20:13. doi:10.5334/ijic.4789.

Smit E, Leenaars KEF, Wagemakers MAE, Molleman GRM, Koelen MA, van der Velden J. Evaluation of the role of Care Sport Connectors in connecting primary care, sport, and physical activity, and residents' participation in the Netherlands: study protocol for a longitudinal multiple case study design. BMC Public Health. 2015;15:510. doi:10.1186/s12889-015-1841-z.

Tenorio-Mucha J, Busta-Flores P, La Cruz-saldana T de, Montufar-Crespo SM, Malaga G, Bernabe-Ortiz A, Lazo-Porras M. Pilot Feasibility Assessment of a Tailored Physical Activity Prescription in Overweight and Obese People in a Public Hospital. Int J Environ Res Public Health 2022. doi:10.3390/ijerph191710774.

Weissenfels A, Klamroth S, Carl J, Naber I, Mino E, Geidl W, Gelius P, Abu-Omar K, Pfeifer K. Effectiveness and implementation success of a co-produced physical activity referral scheme in Germany: study protocol of a pragmatic cluster randomised trial. BMC Public Health. 2022 Aug 13;22(1):1545. doi: 10.1186/s12889-022-13833-2

Woods C, McCaffrey N, Furlong B, Fitzsimons-D’Arcy, L, Murphy, M, Harrison M, Glynn L, et al. The National Exercise Referral Framework. Ireland: Dublin; 2016.
